# Supplementary material for: Synthesis of Pluri-Functional Amine Hardeners from Bio-Based Aromatic Aldehydes for Epoxy Amine Thermosets
Source: Molecules. 2019 Sep 9;24(18):3285. doi: 10.3390/molecules24183285 (PMC6766844; doi:10.3390/molecules24183285)

Supporting Information

Synthesis of pluri-functional amine hardeners from bio-based aromatic aldehydes for epoxy amine thermosets

Anne-Sophie Mora[a], Russell Tayouo[b], Bernard Boutevin[a], Ghislain David[a], Sylvain Caillol[a*]

a) Institut Charles Gerhardt, UMR 5253 – CNRS, Université de Montpellier, Ecole Nationale Supérieure de Chimie de Montpellier, 240 Avenue Emile Jeanbrau, 34296 Montpellier, France
b) SAS Nouvelle Sogatra, 784 Chemin de la caladette, 30350 Lezan, France
E-mail: sylvain.caillol@enscm.fr; Fax: +33-467-14-72-20; Tel: +33-467-14-43-27.

Table of Contents

1. Synthesis from vanillin 3
   a. Imine synthesis 3
2. Synthesis from 3,4-dihydroxybenzaldehyde 4
   a. Reaction crude of imine synthesis 4
   b. Reaction crude of imine reduction 4
3. IPTA characterizations 5
   a. Imine 5
   b. Amine 6
4. BDA characterizations 9
   a. Imine 9
   b. Amine 12
5. FDA characterizations 15
   a. Imine 15
   b. Amine 18
6. TGA measurements of each synthesized amines (under nitrogen, at 20 $K.min^{-1}$) 21
7. DSC measurements of each synthesized amines (under nitrogen, at 20 K.min-1) 21
8. Determination of optimal ratio for partially bio-based thermosets 22
   a. From IPTA1 and DGEBA 22
   b. From IPT2 and DGEBA 22
   c. From BDA and DGEBA 23
   d. From FDA and DGEBA 23
9. Determination of optimal ratio for partially bio-based thermosets 24
   a. From IPTA1 and DGEVA 24
   b. From IPTA2 and DGEVA 24
   c. From BDA and DGEVA 25
   d. From FDA and DGEVA 25
10. Characterizations of each bulk materials synthesized with optimal ratio 26
   a. DSC of partially bio-based thermosets 26
   b. DSC of fully bio-based thermosets 26
   c. FT-IR of partially bio-based thermosets 27

1. IPTA characterizations
   a. Imine
      i. FT-IR

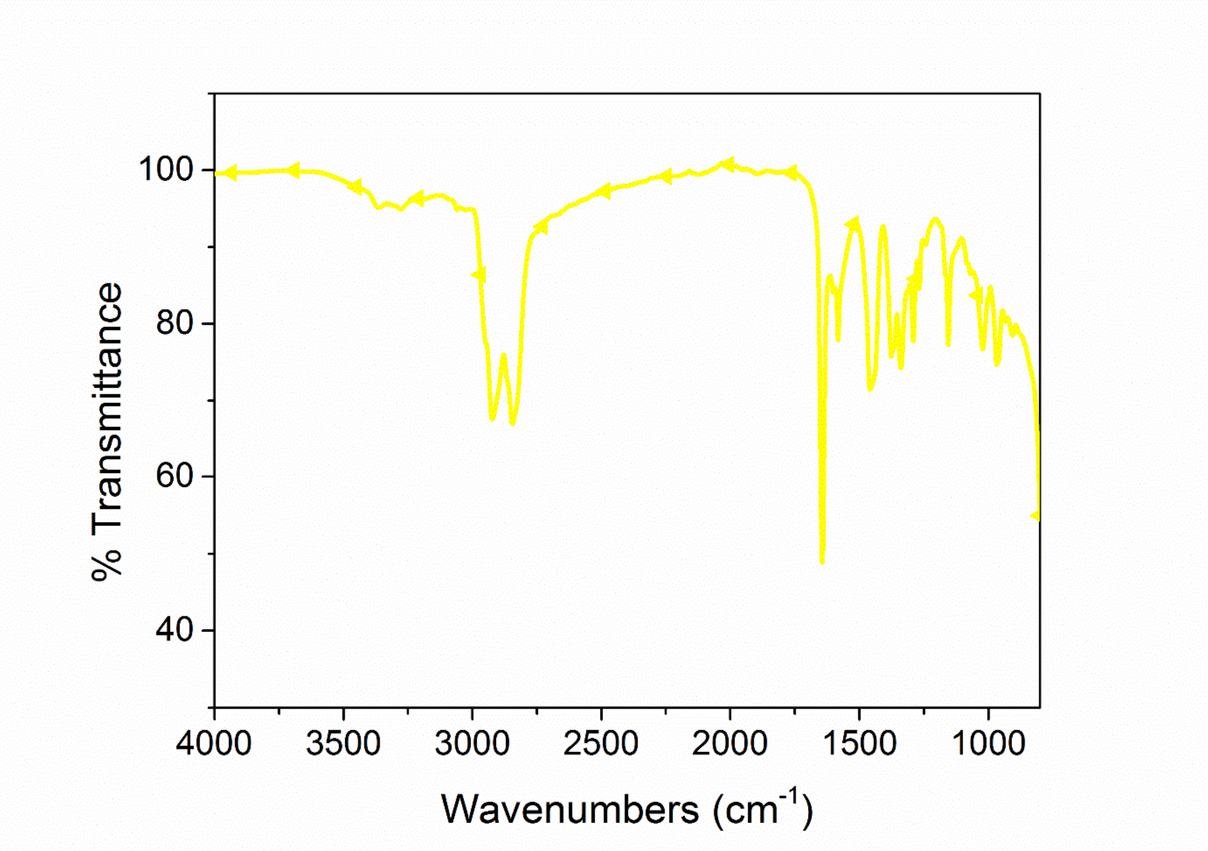

      ii. ^{1}H NMR

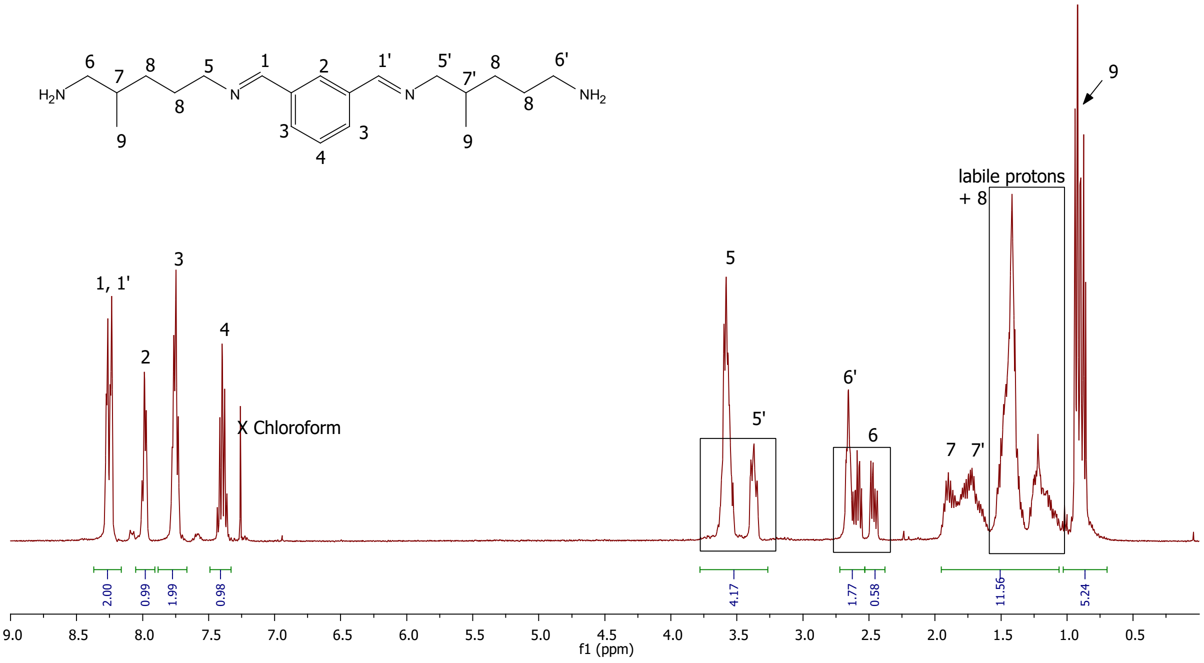

iii. $^{1}H - ^{1}H$ COSY NMR

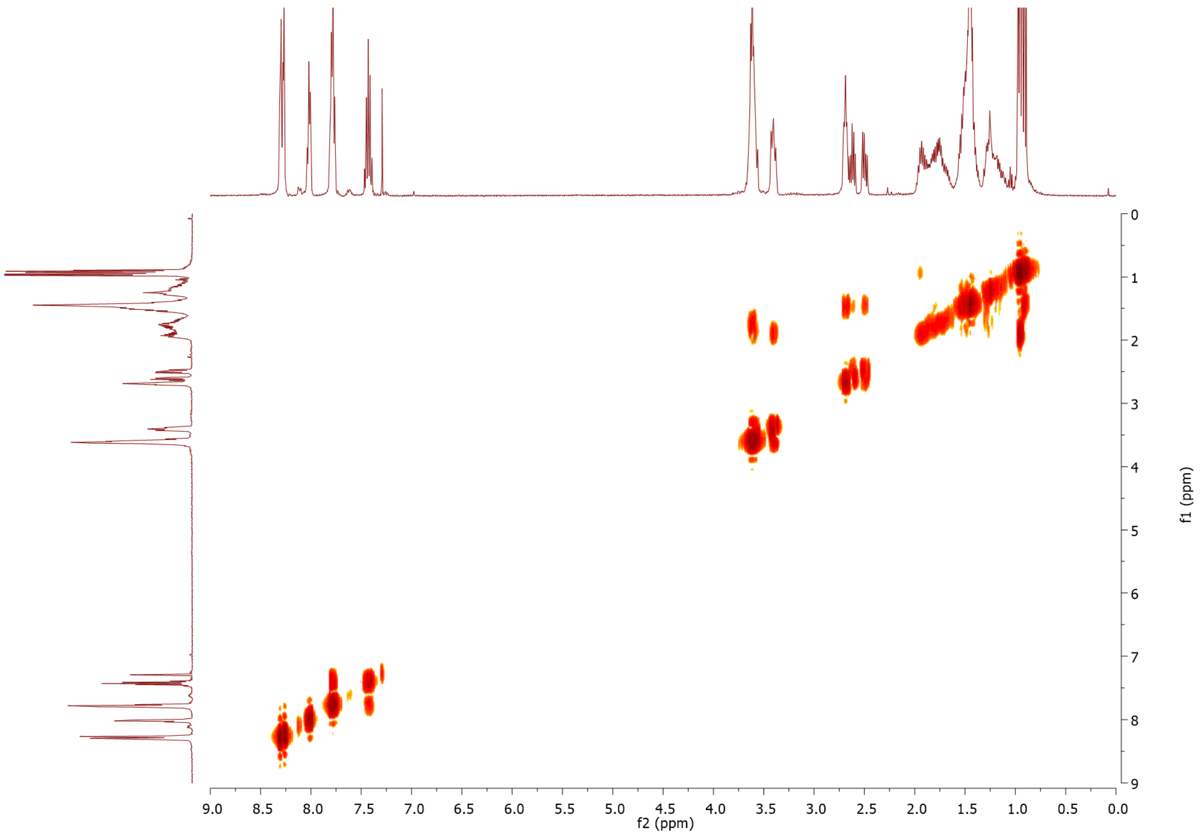

iv. ^{13}C (up) and DEPT 135 (down) NMR

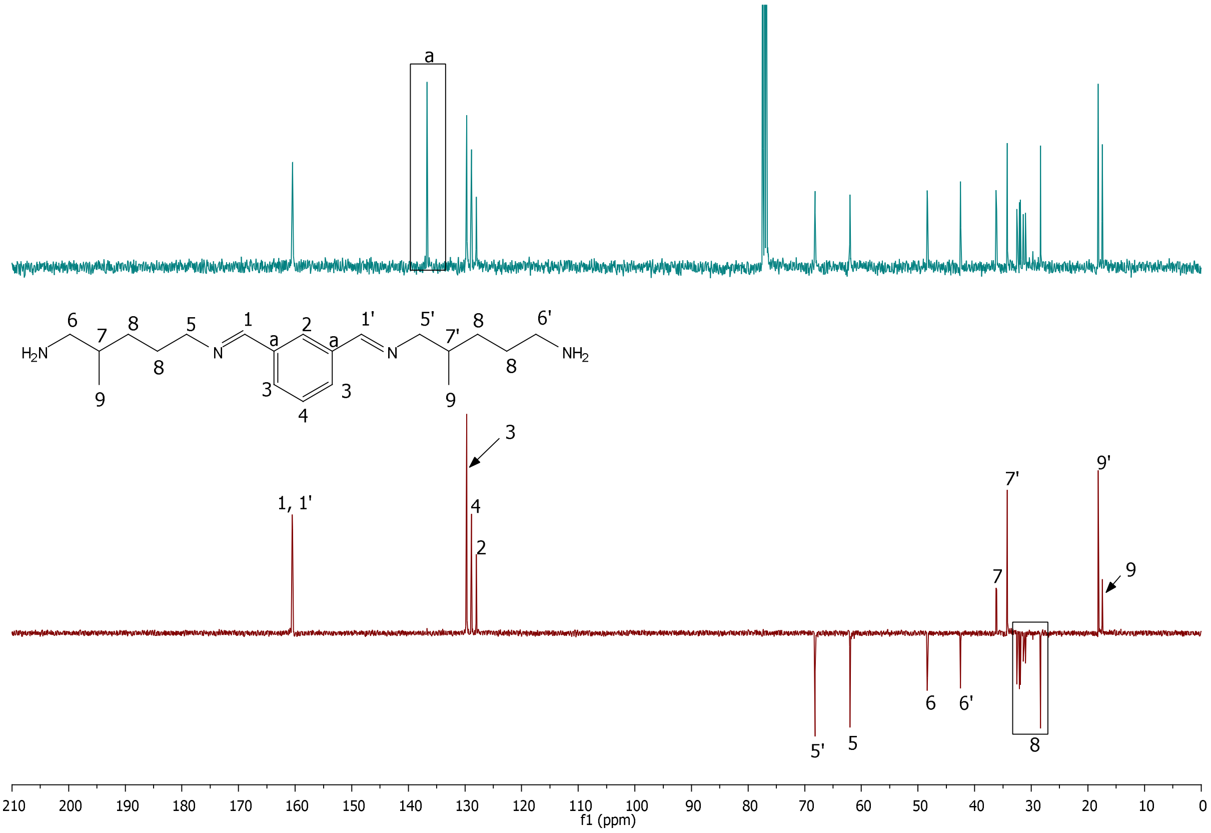

v. ^{1}H – ^{13}C HSQC NMR

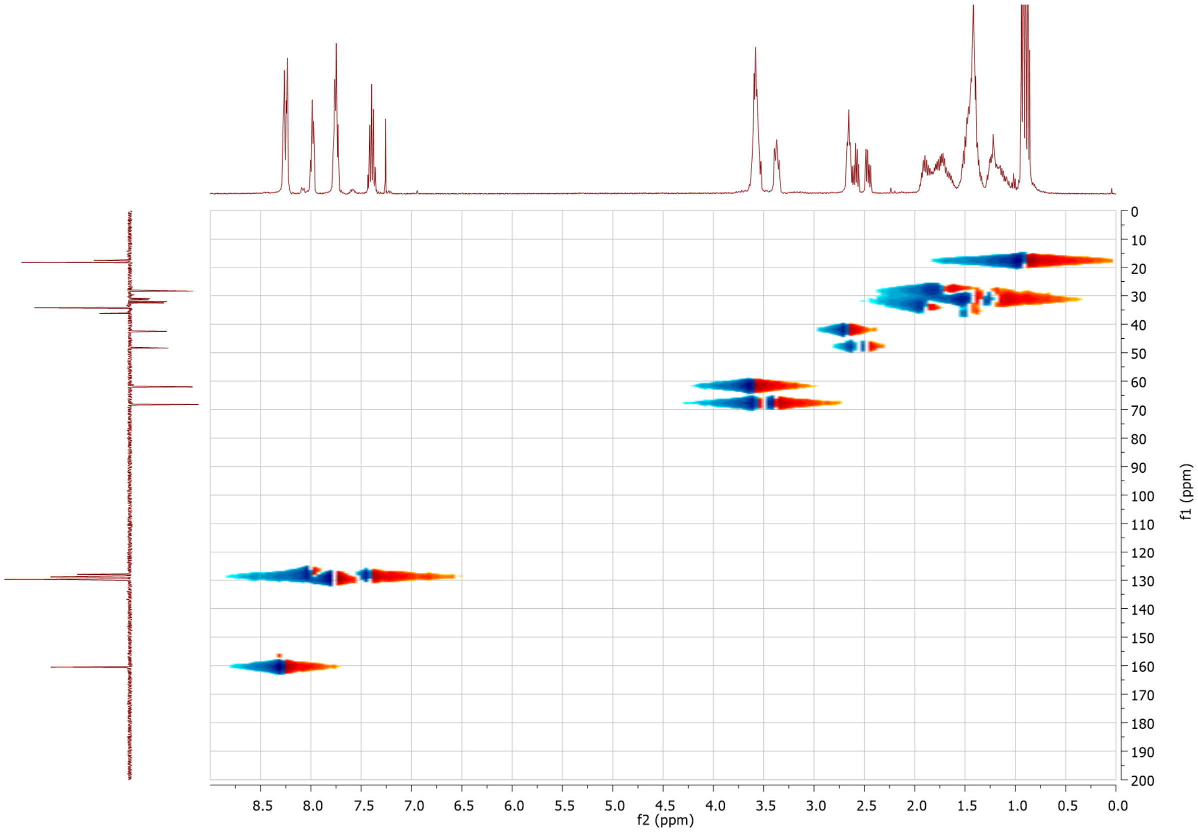

b. Amine

i. FT-IR

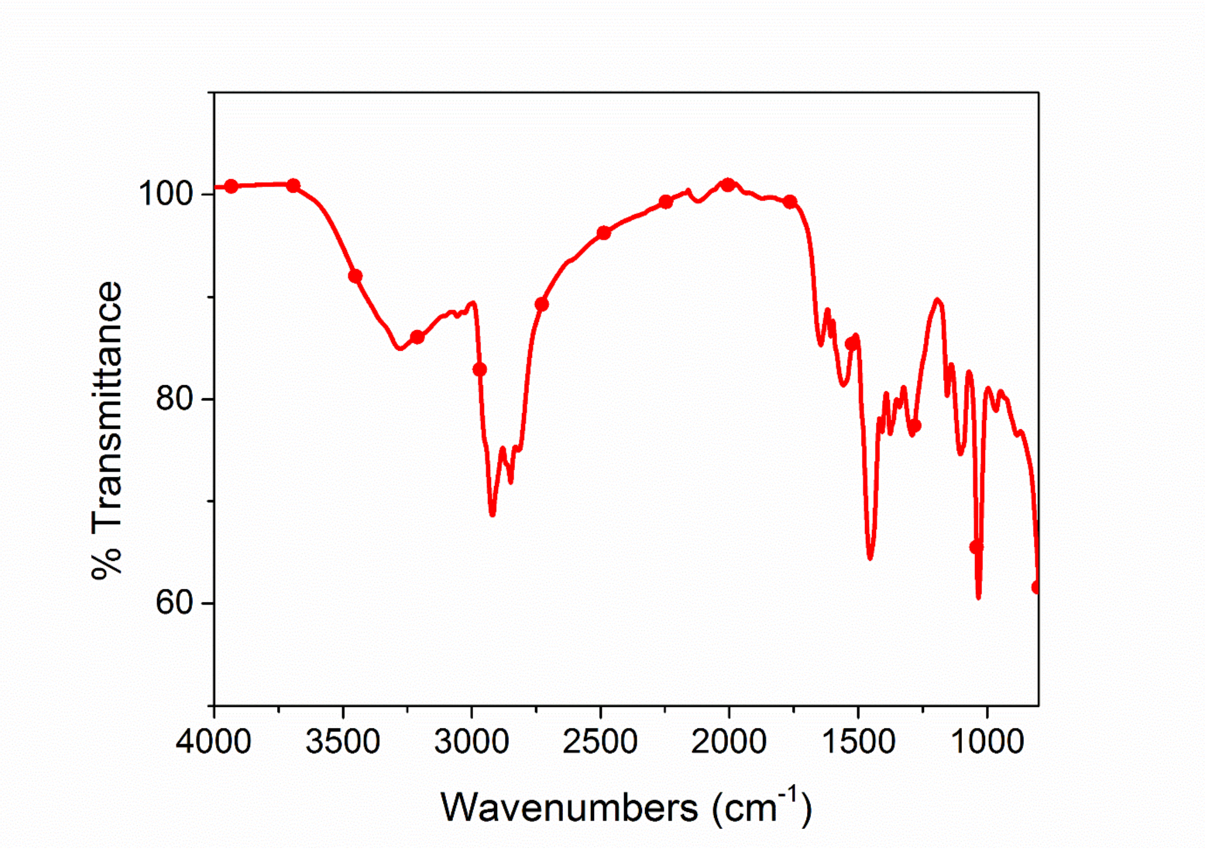

ii. ^{1}H NMR

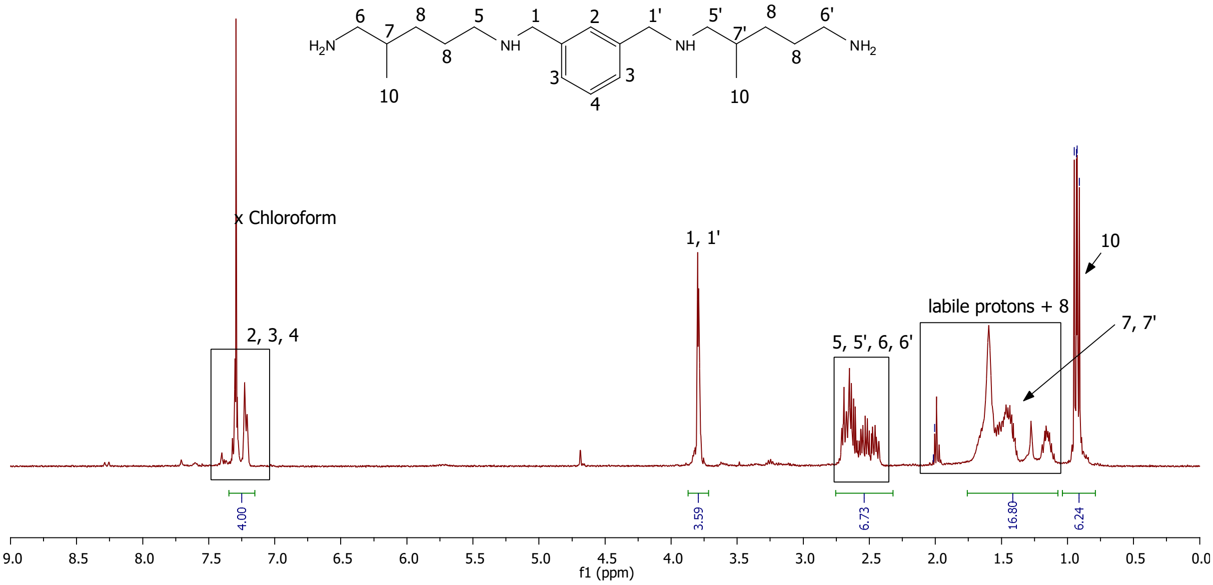

iii. $^{1}H - ^{1}H$ COSY NMR

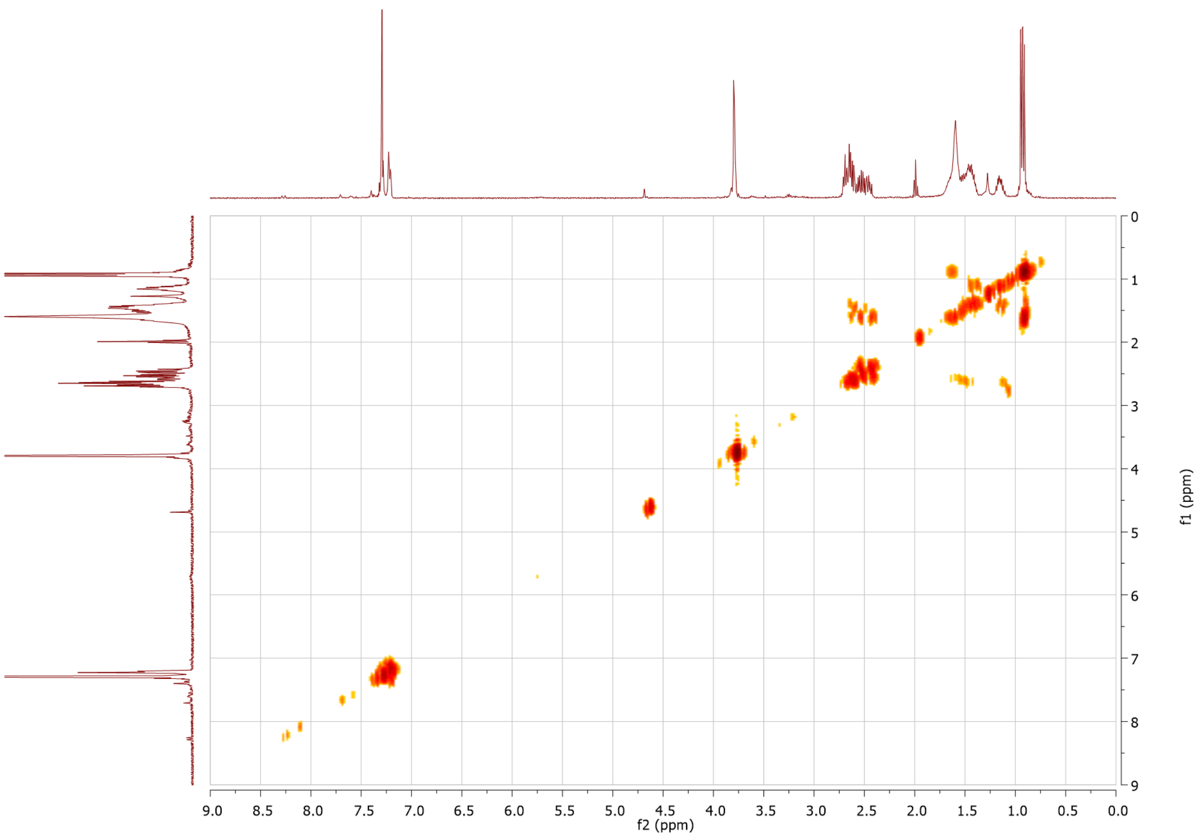

iv. ^{13}C (up) and DEPT 135 (down) NMR

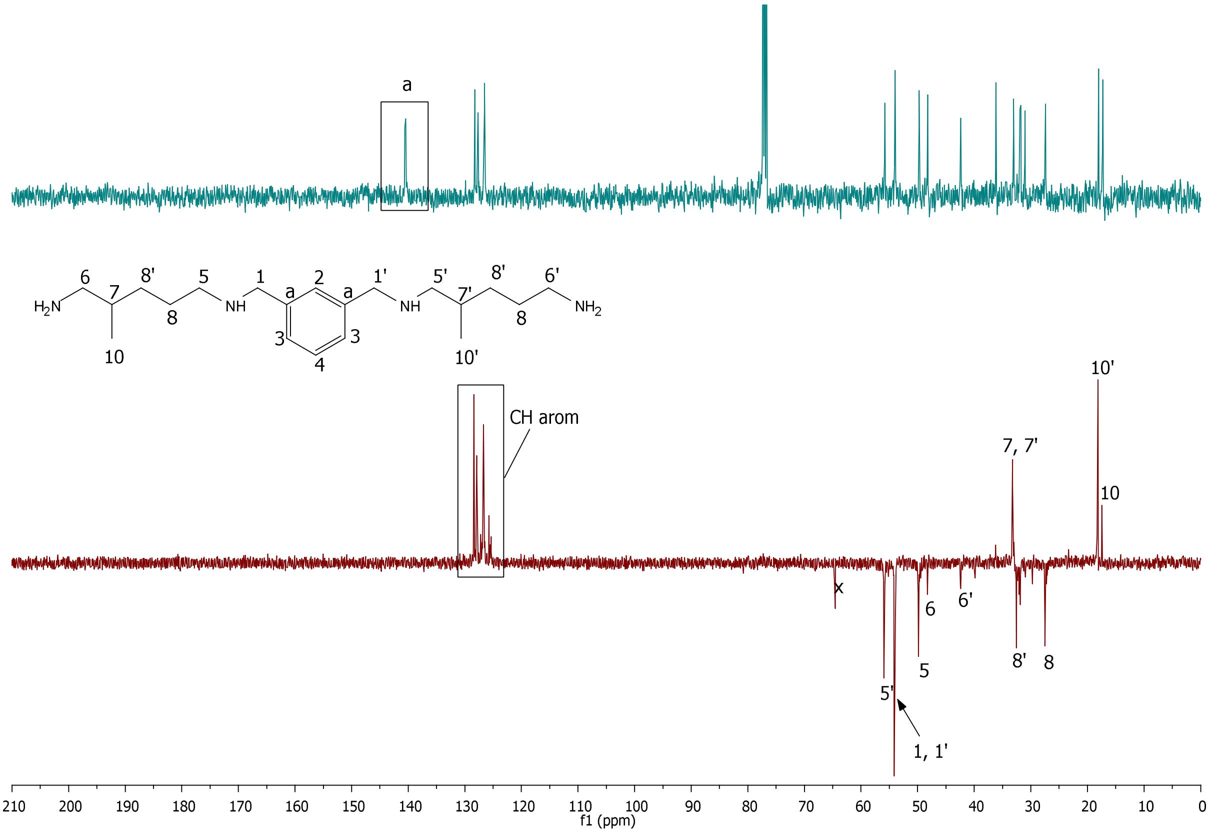

v. $^{1}H - ^{13}C$ HSQC NMR

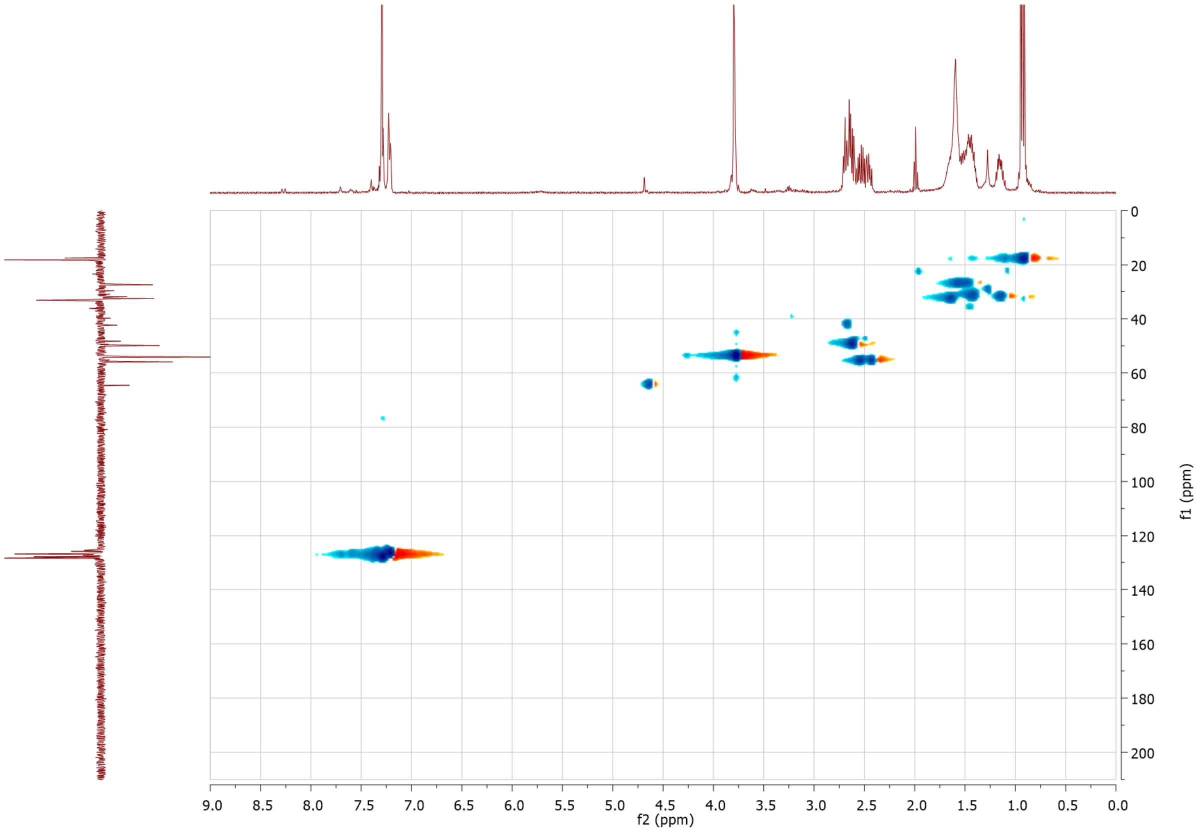

2. BDA characterizations

a. Imine

i. FT-IR

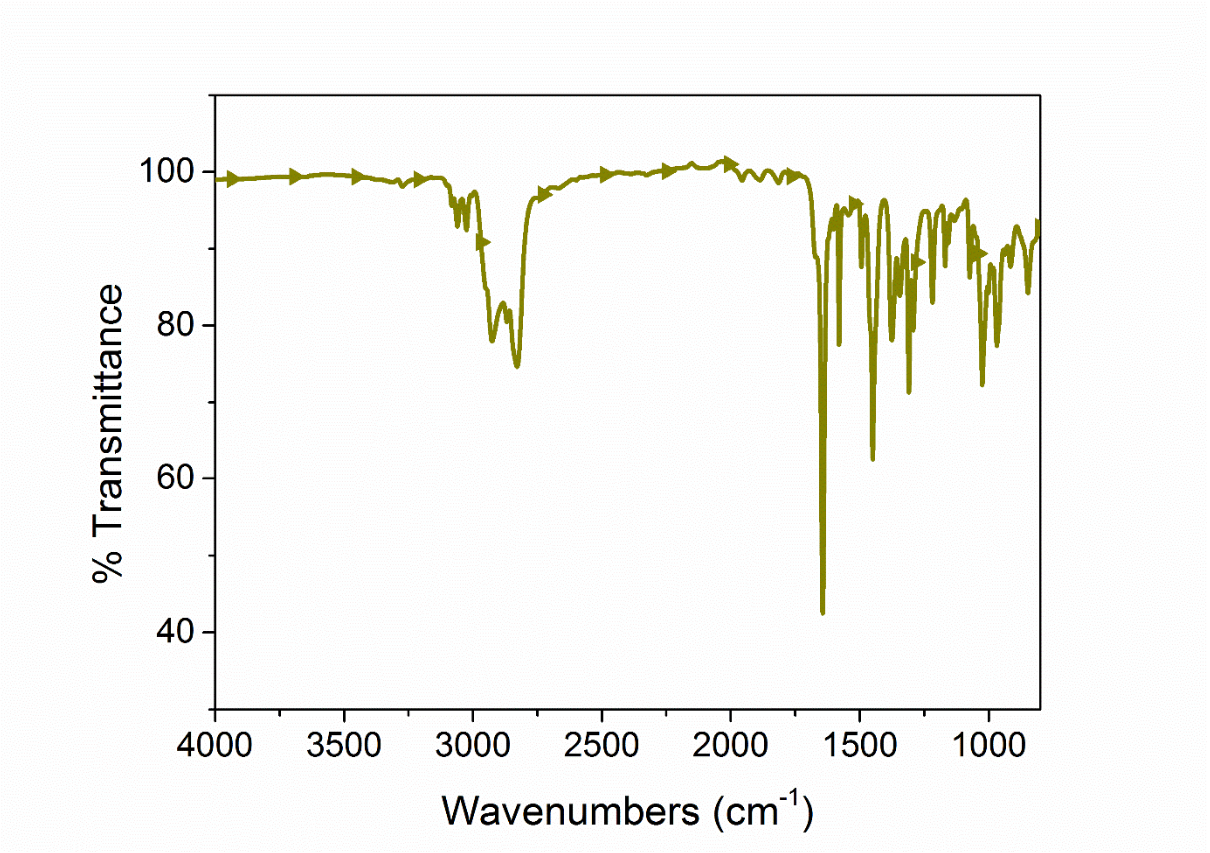

ii. ^{1}H NMR

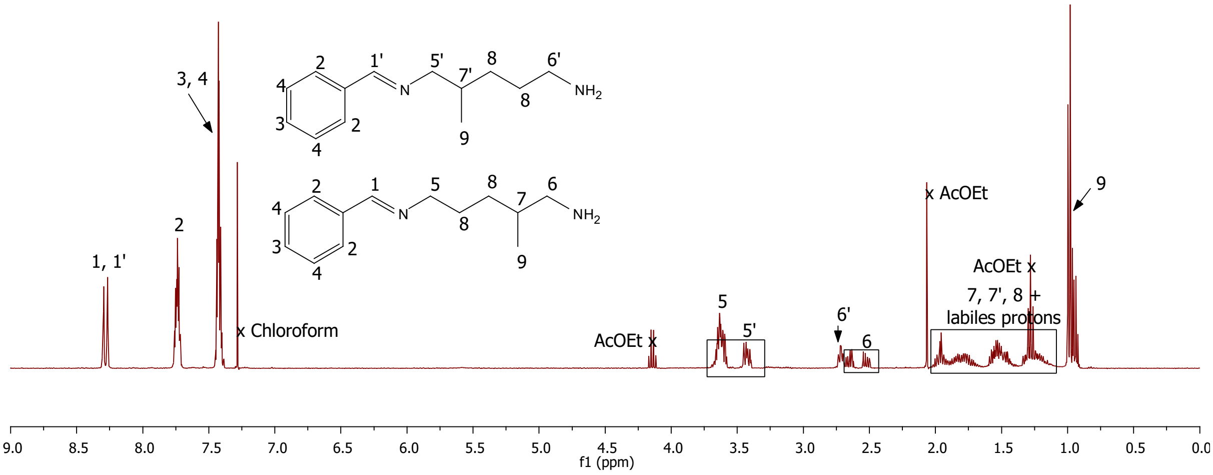

iii. $^{1}H - ^{1}H$ COSY NMR

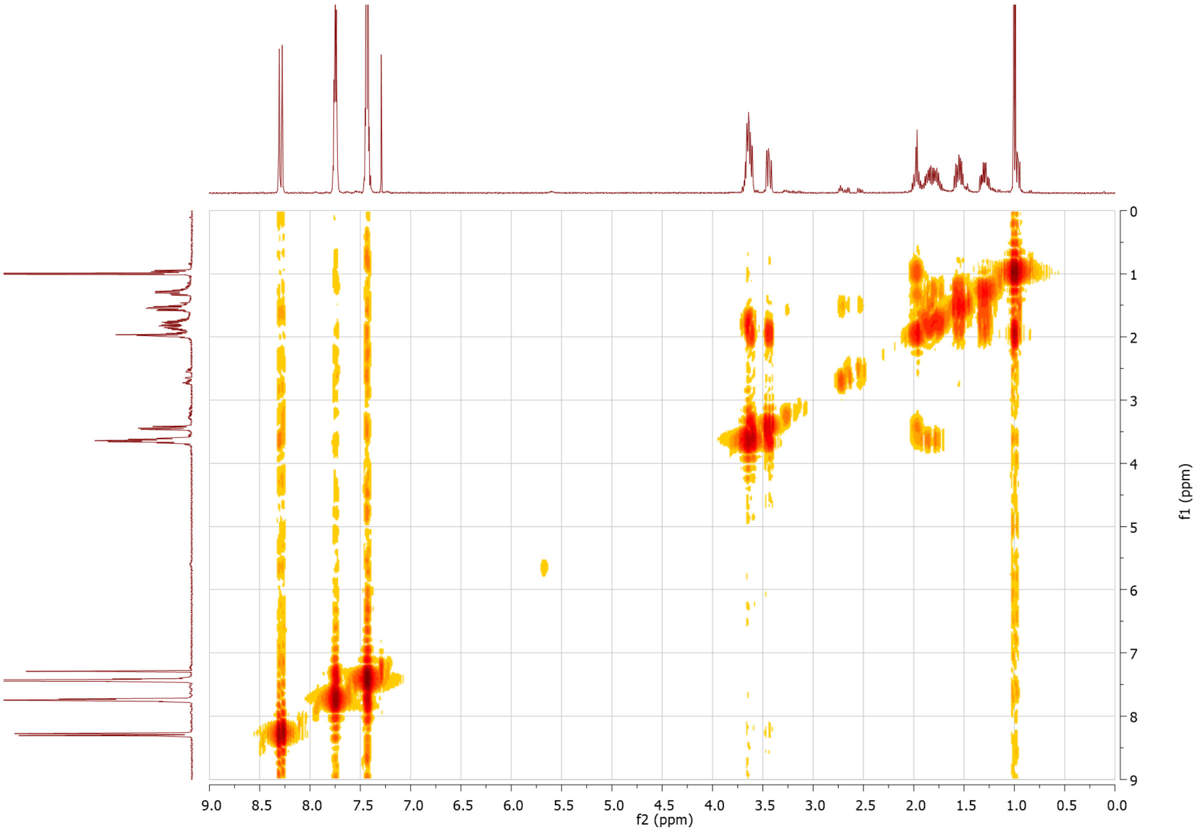

iv. ^{13}C (up) and DEPT 135 (down) NMR

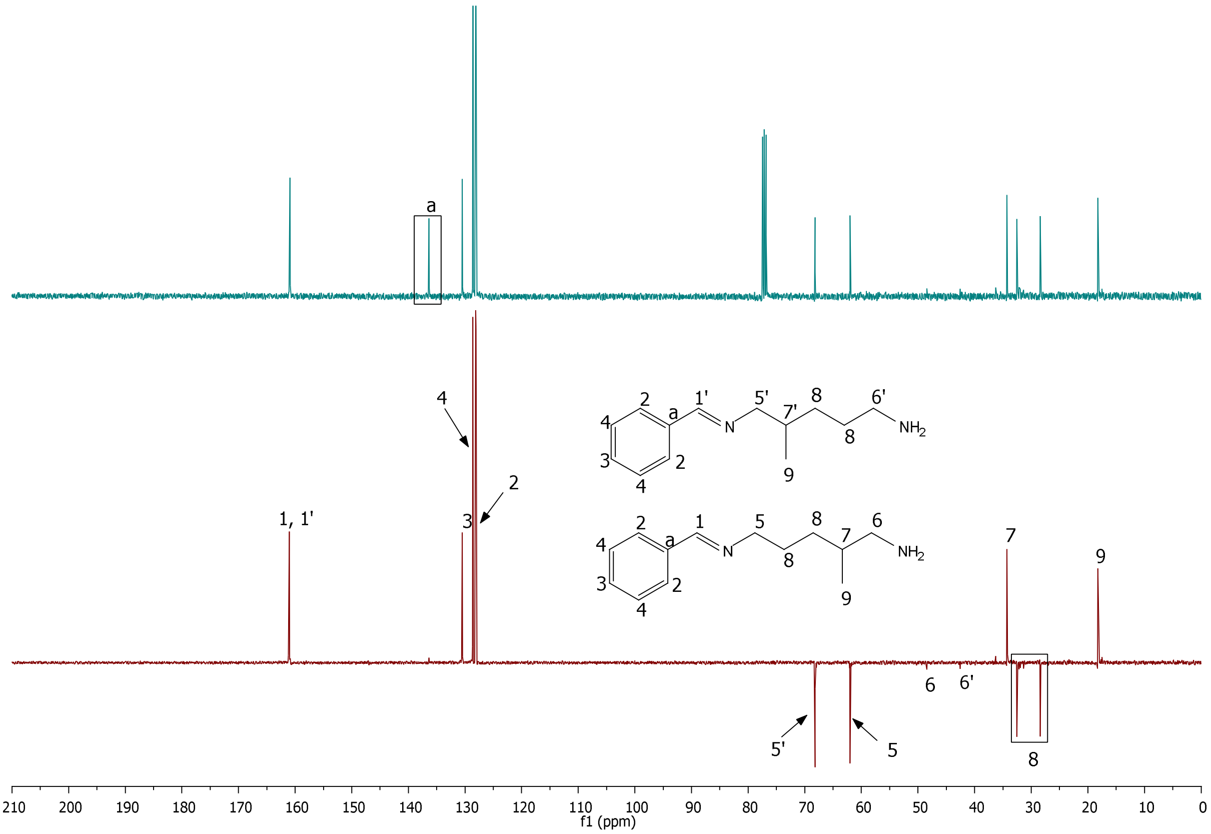

v. $^{1}H - ^{13}C$ HSQC NMR

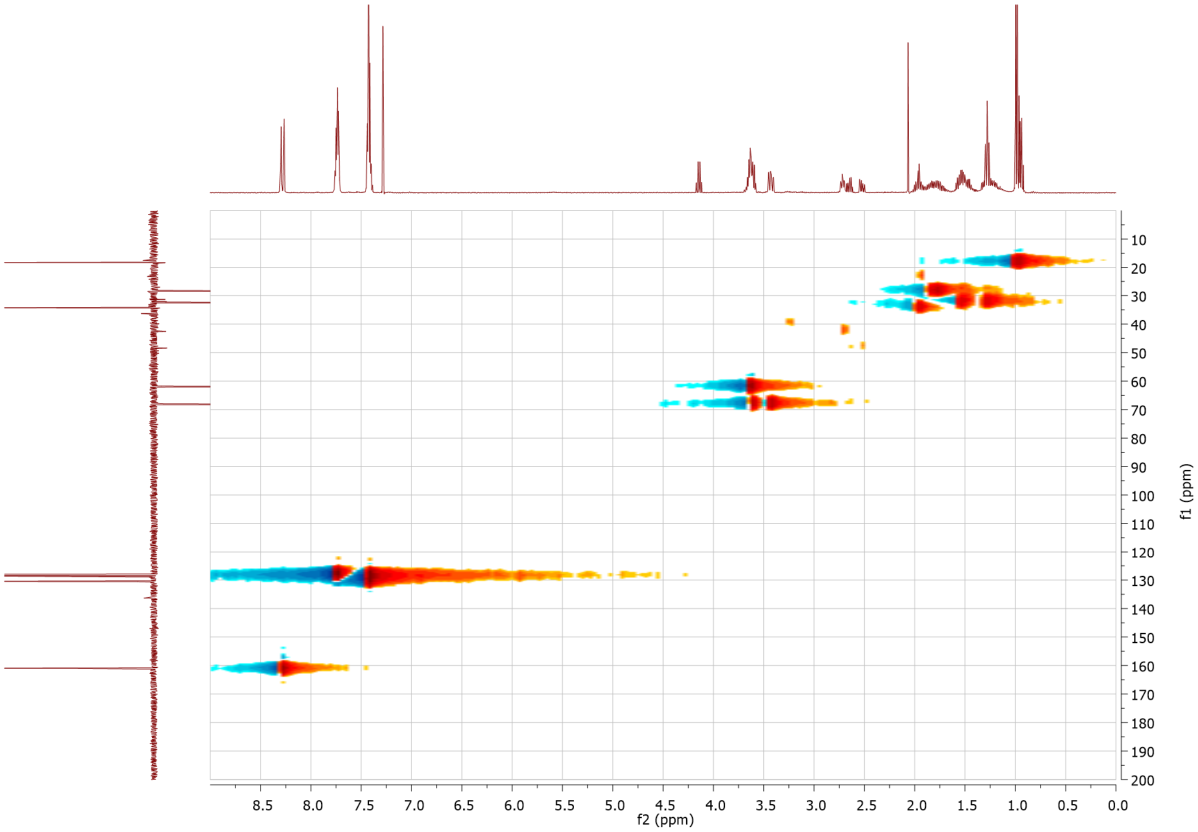

b. Amine

i. FT-IR

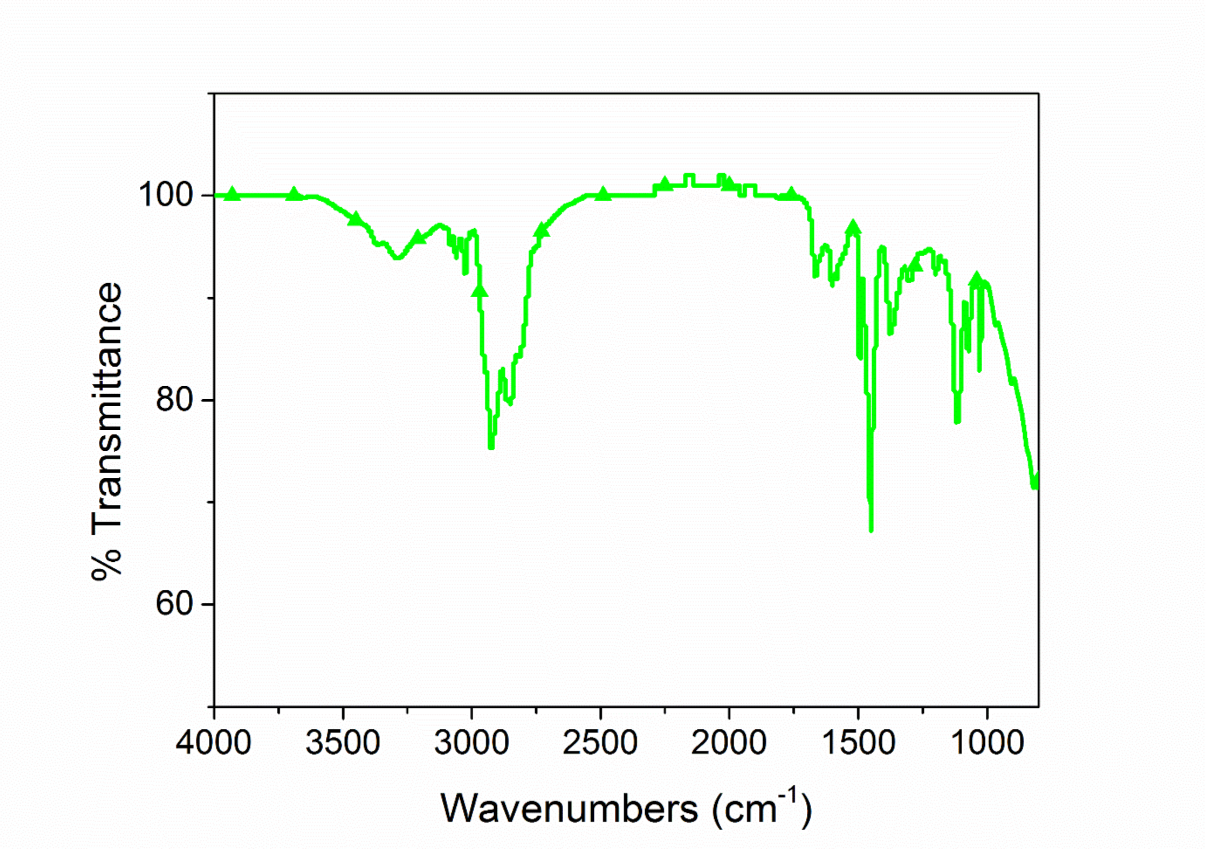

ii. ^{1}H NMR

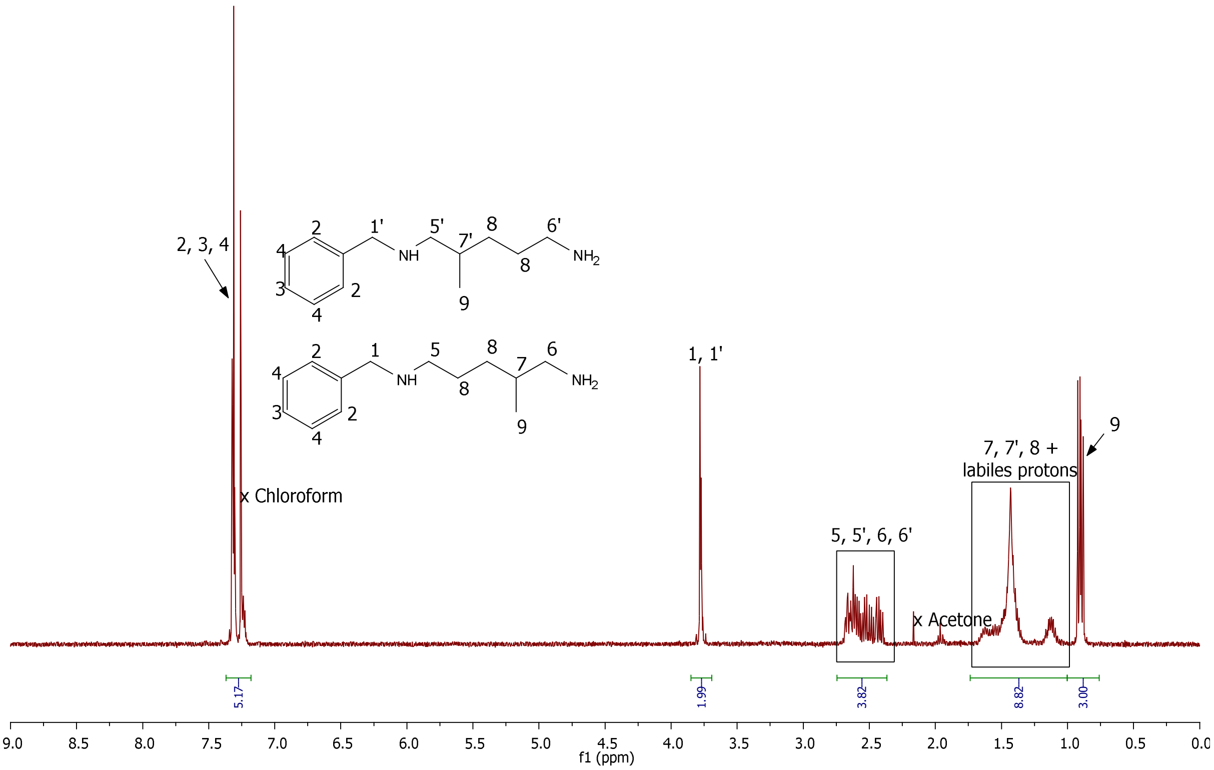

iii. $^1H - ^1H$ COSY NMR

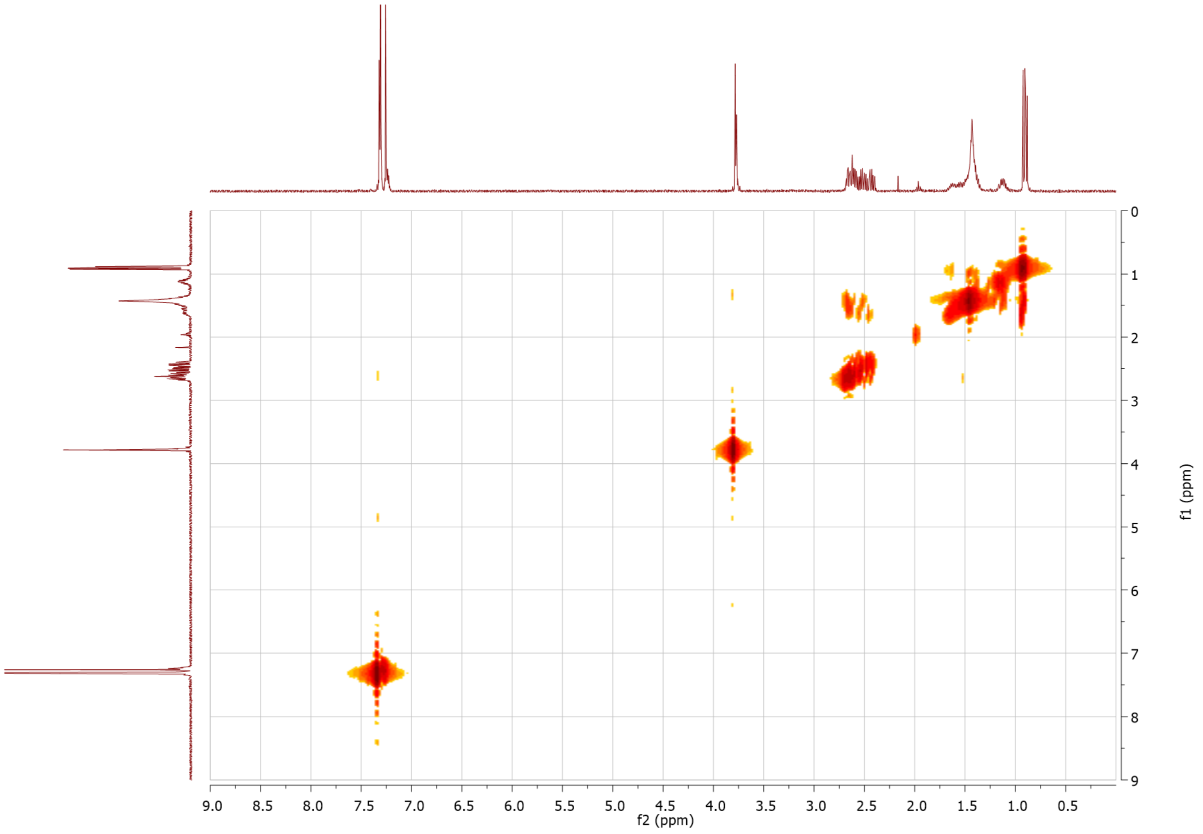

iv. ^{13}C (up) and DEPT 135 (down) NMR

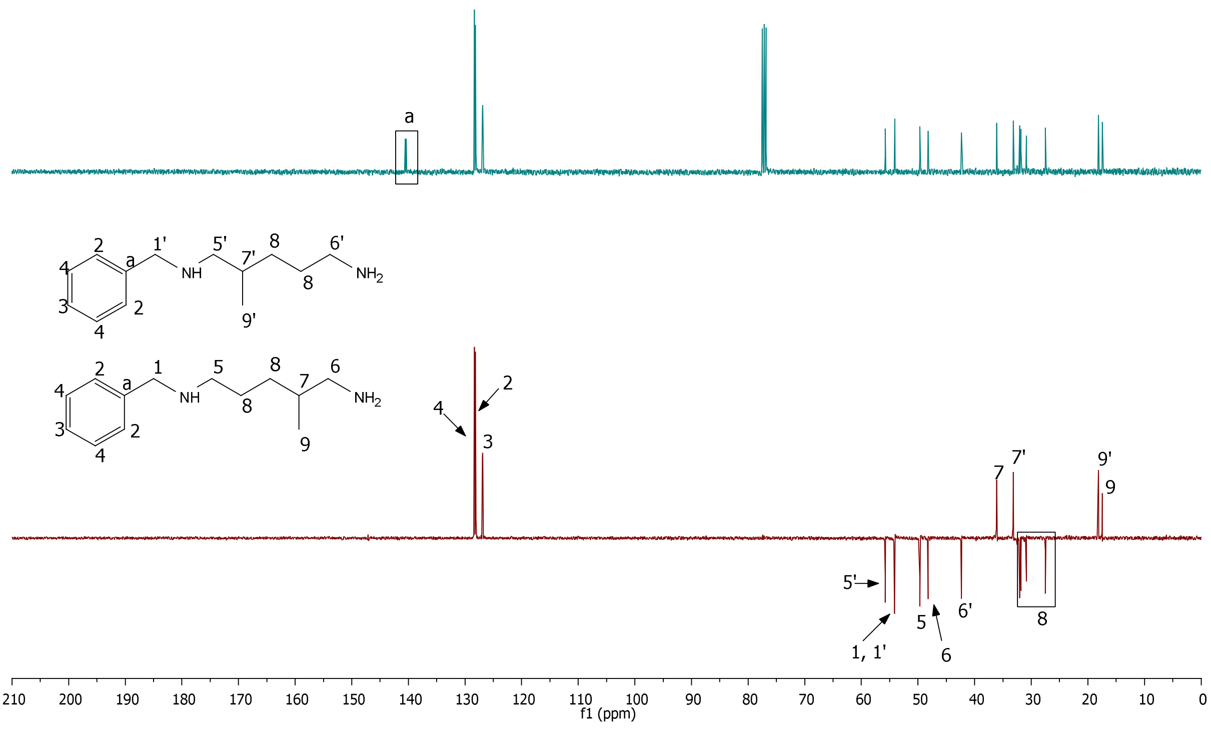

v. $^{1}H - ^{13}C$ HSQC NMR

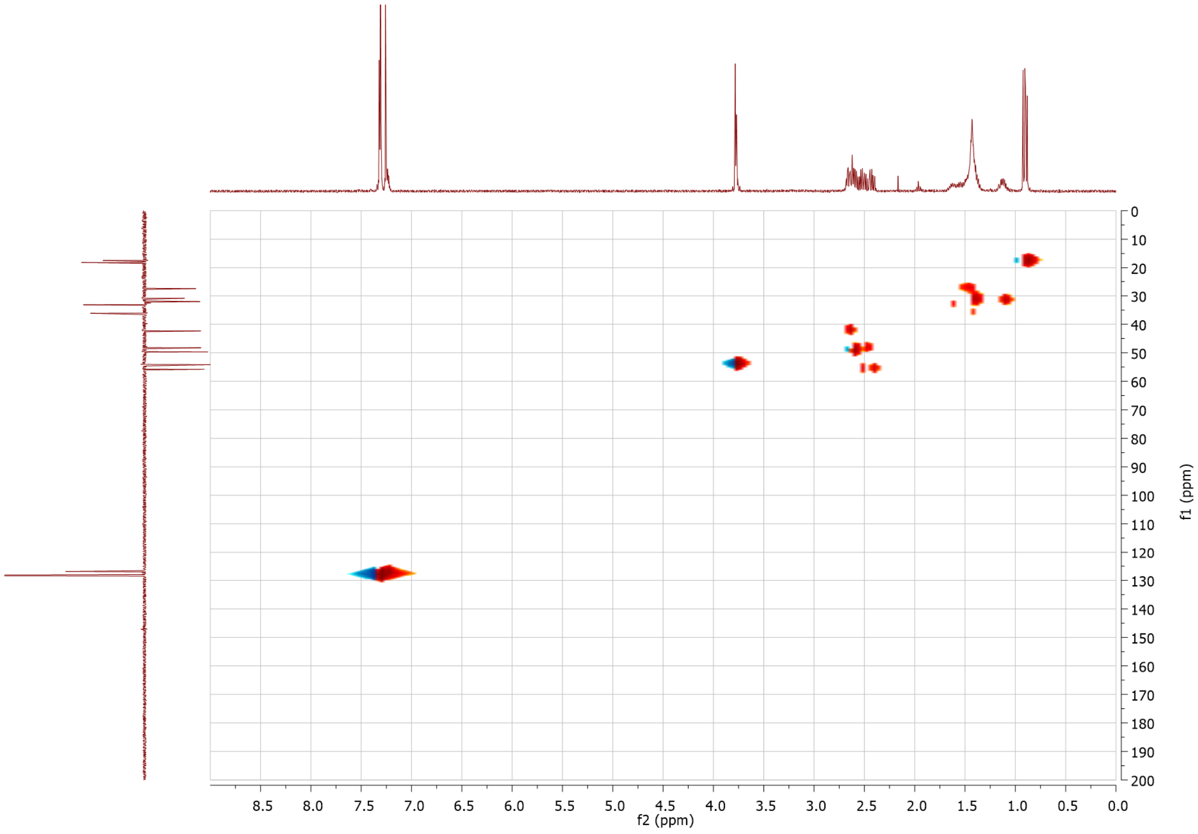

3. FDA characterizations

a. Imine

i. FT-IR

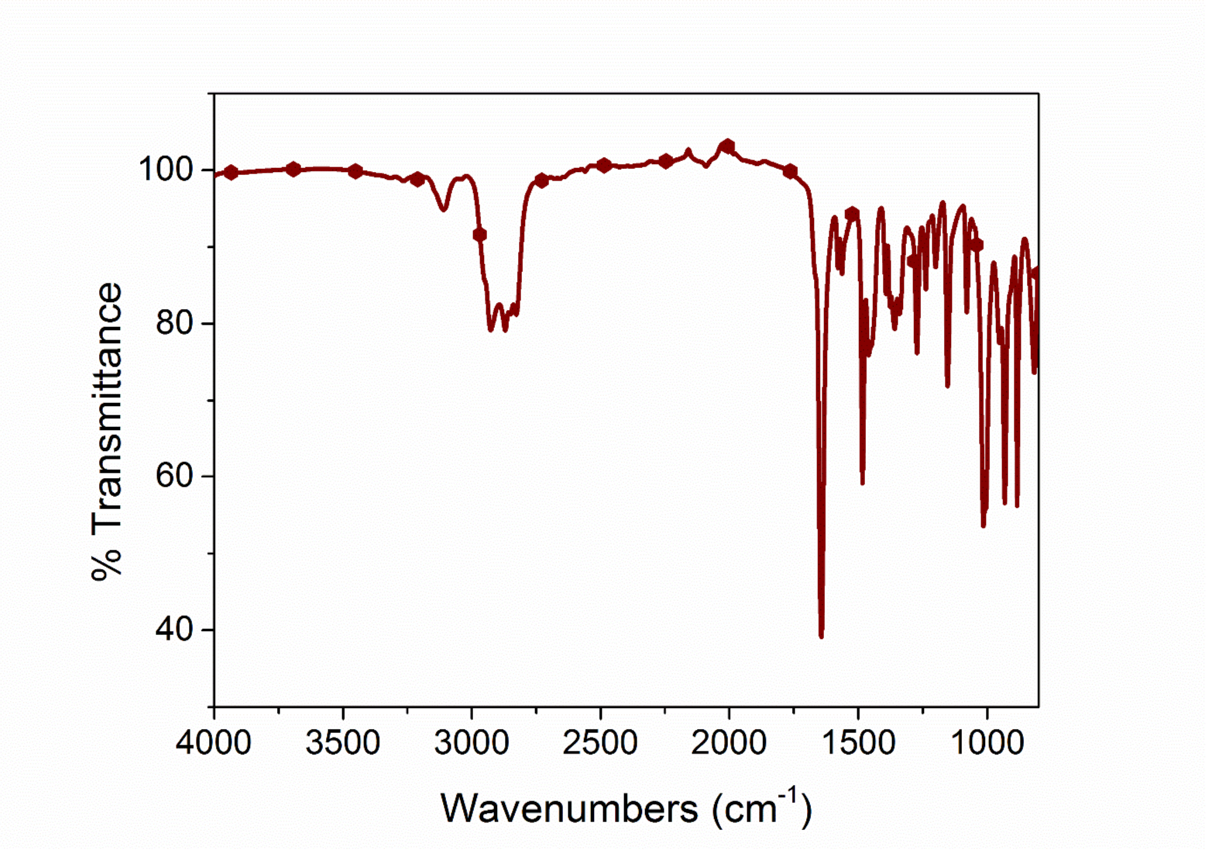

ii. ^{1}H NMR

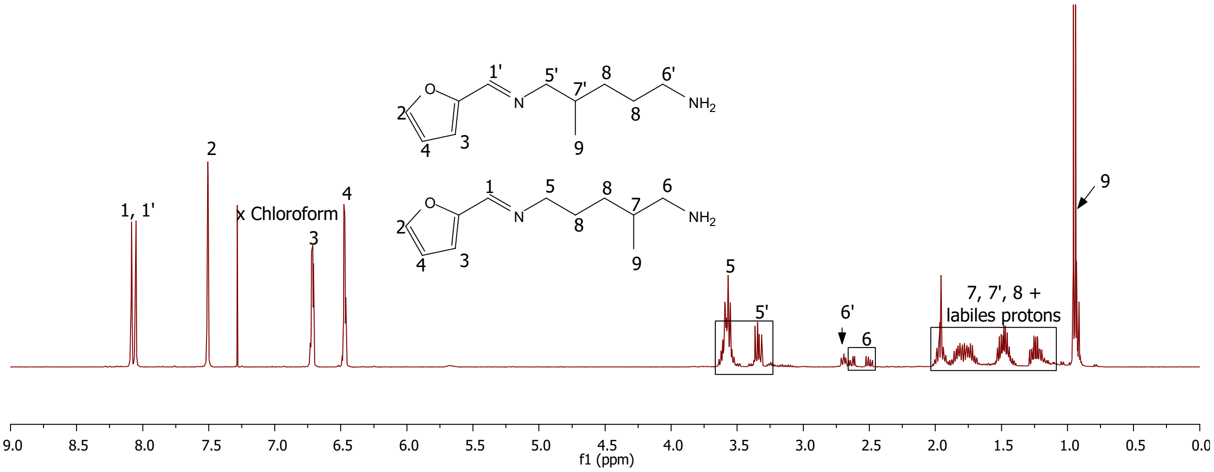

iii. $^{1}H - ^{1}H$ COSY NMR

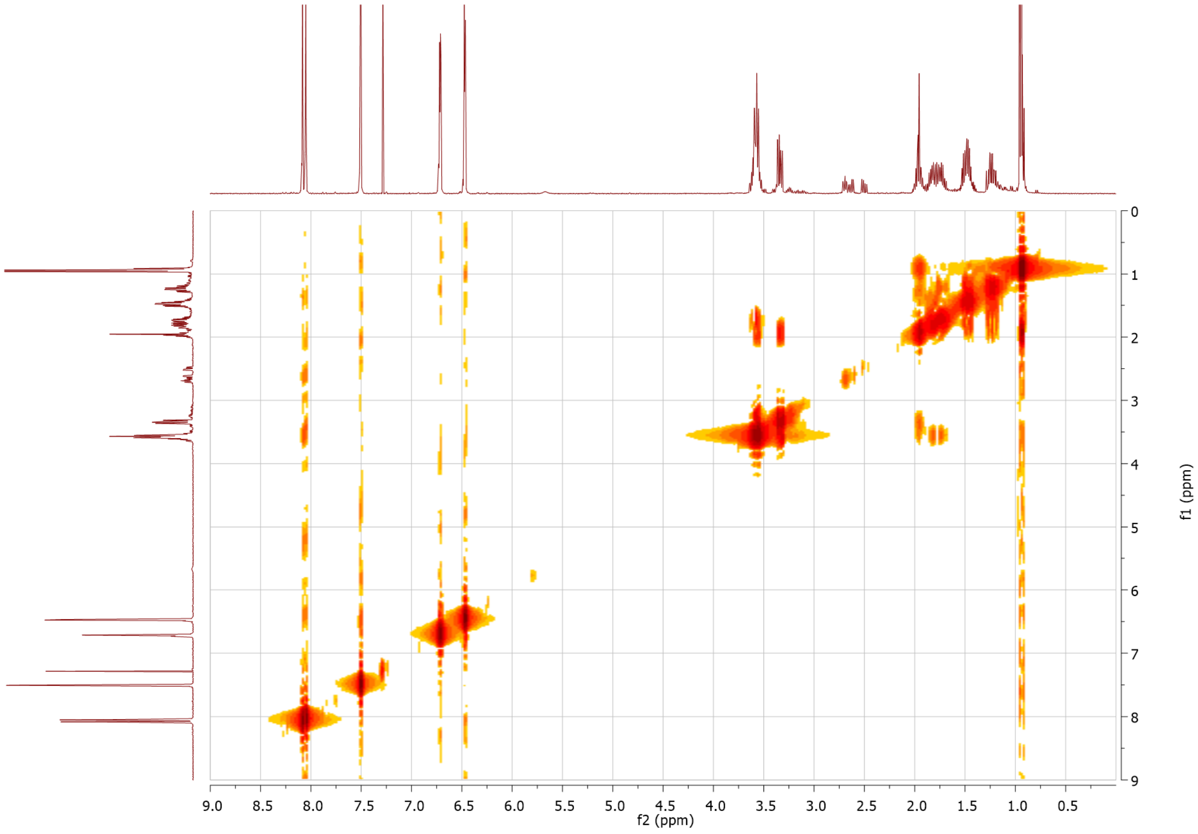

iv. ^{13}C (up) and DEPT 135 (down) NMR

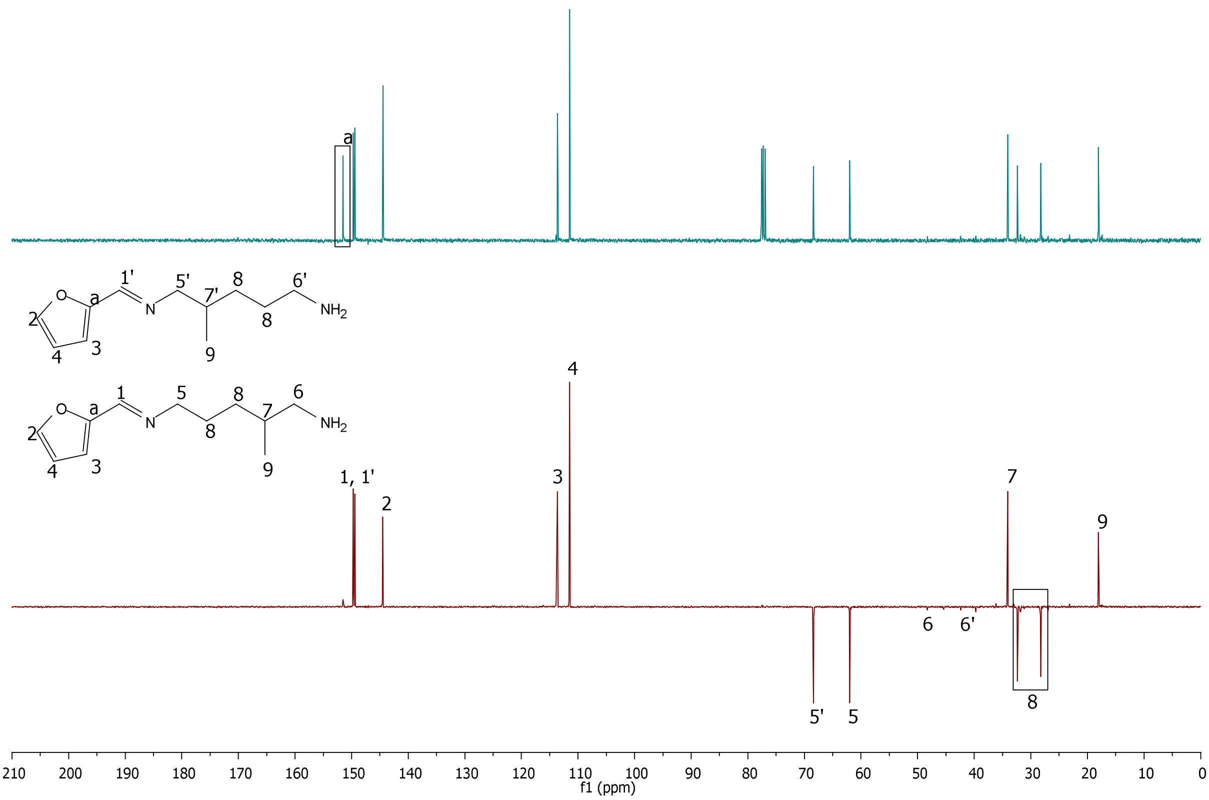

v. $^{1}H - ^{13}C$ HSQC NMR

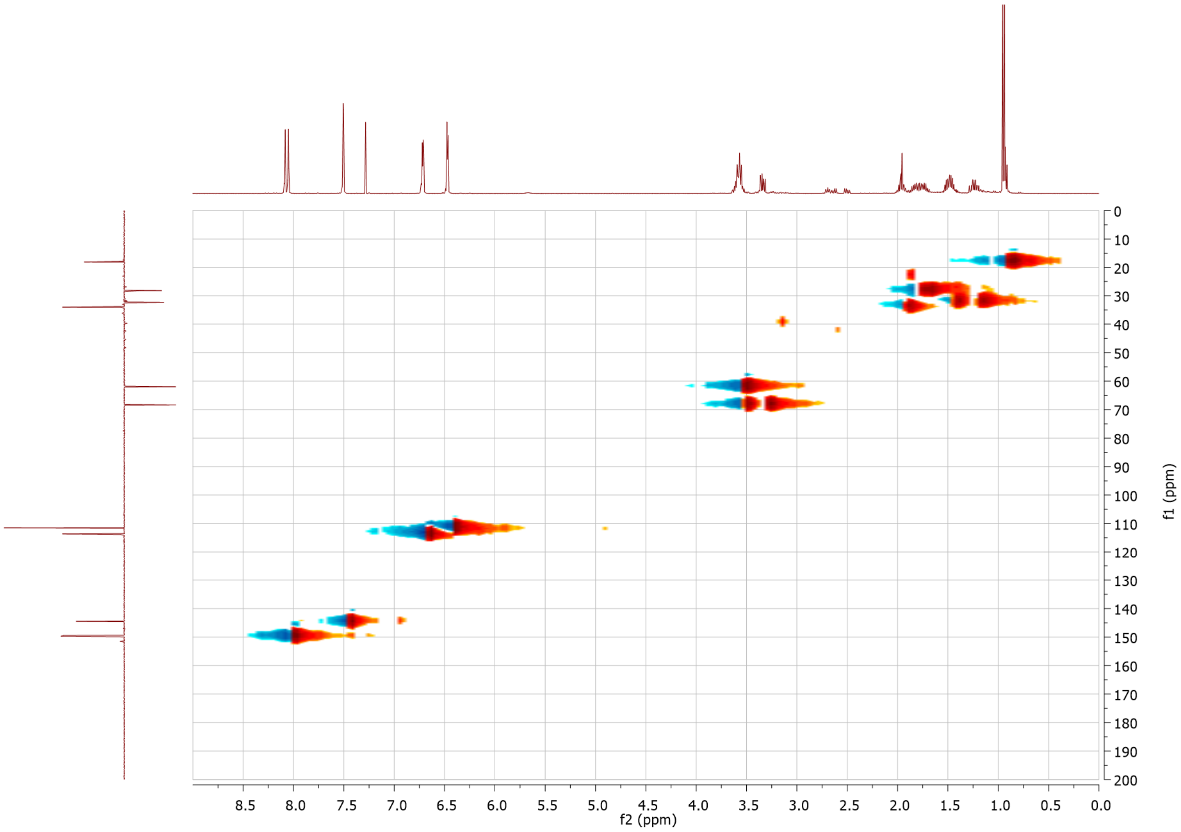

b. Amine

i. FT-IR

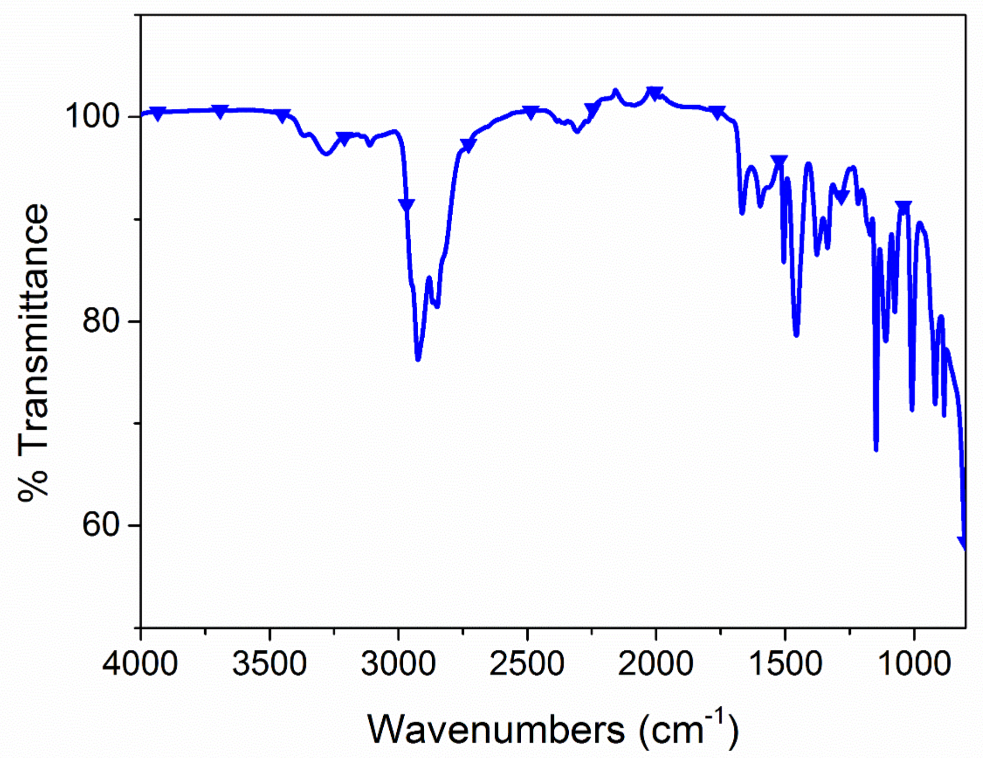

ii. ^{1}H NMR

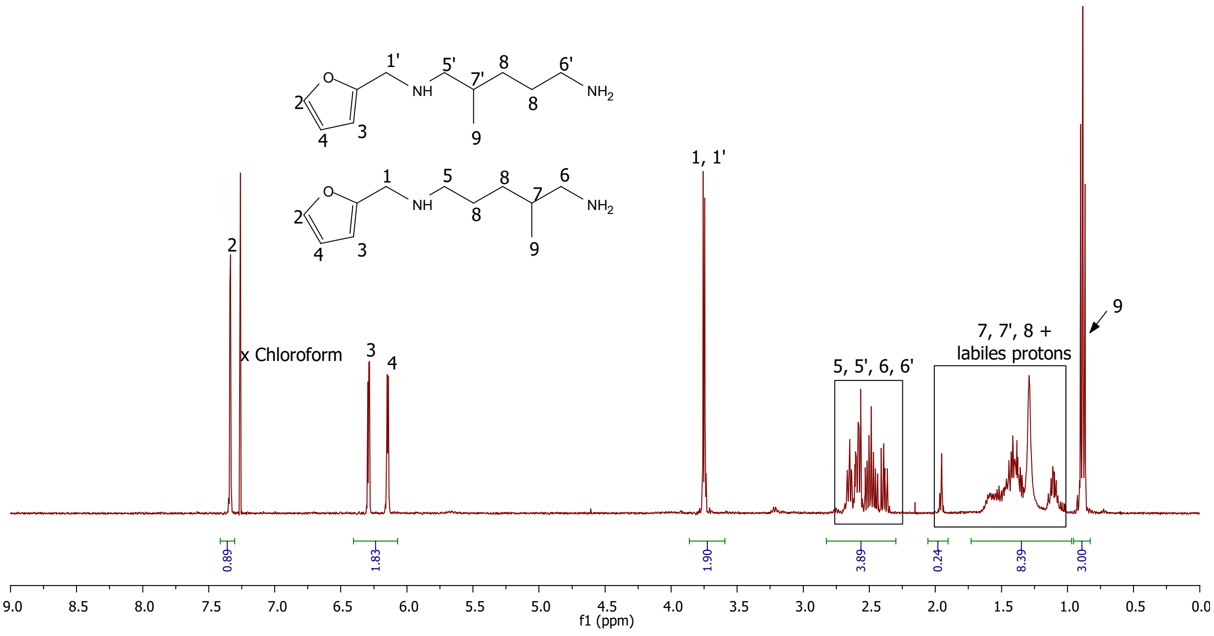

iii. ^{1}H – ^{1}H COSY NMR

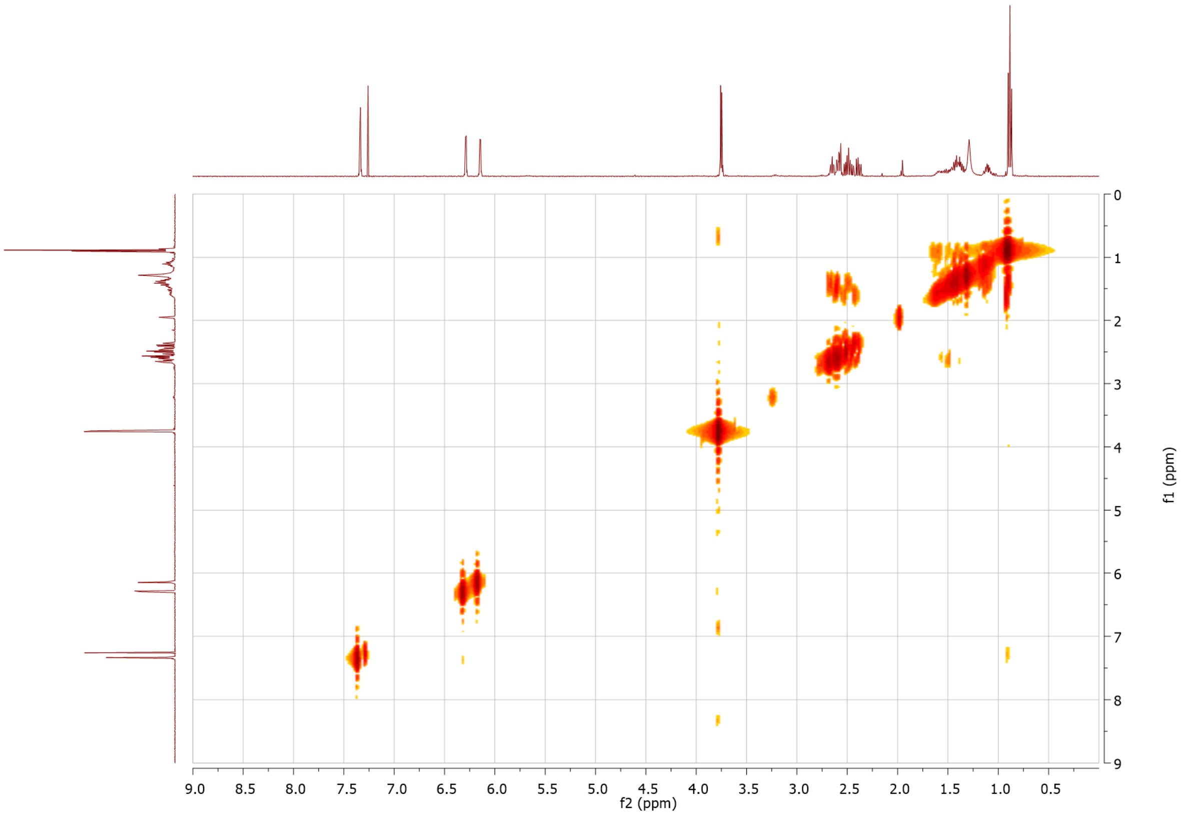

iv. ^{13}C (up) and DEPT 135 (down) NMR

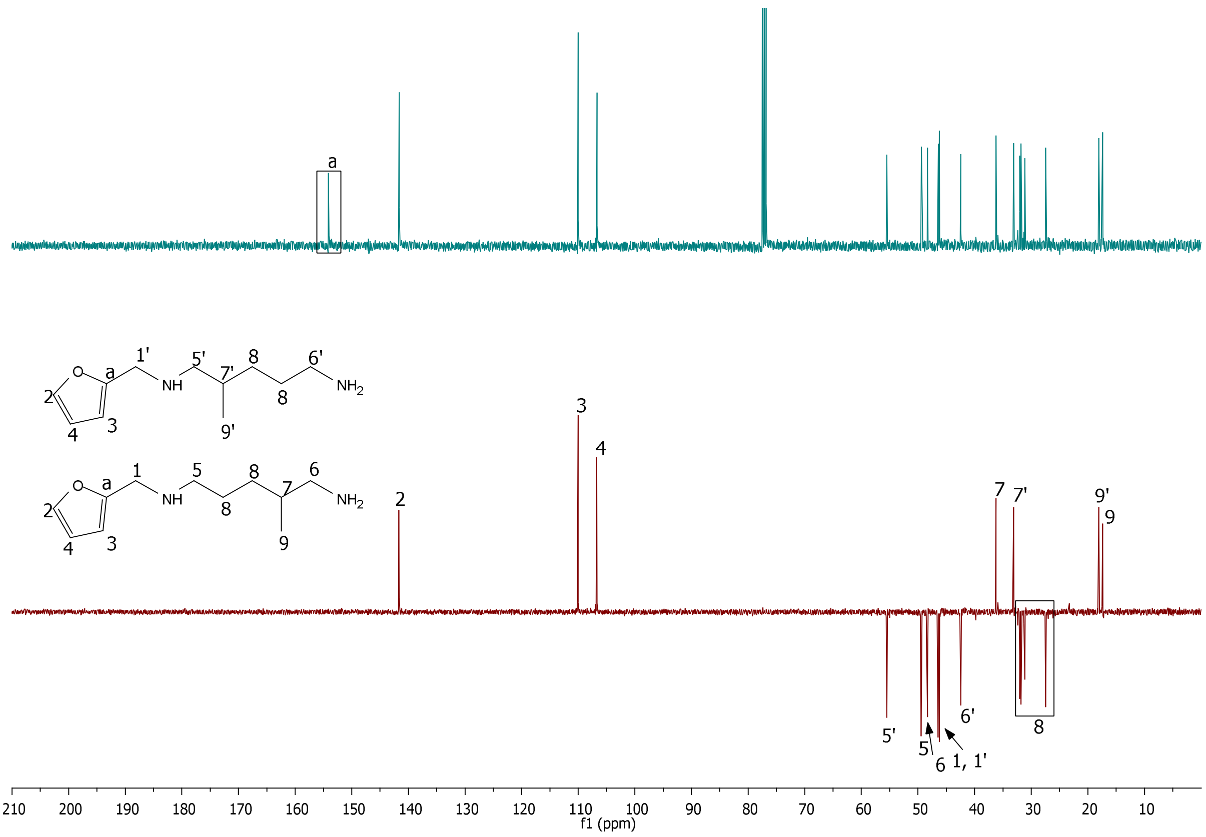

v. $^{1}H - ^{13}C$ HSQC NMR

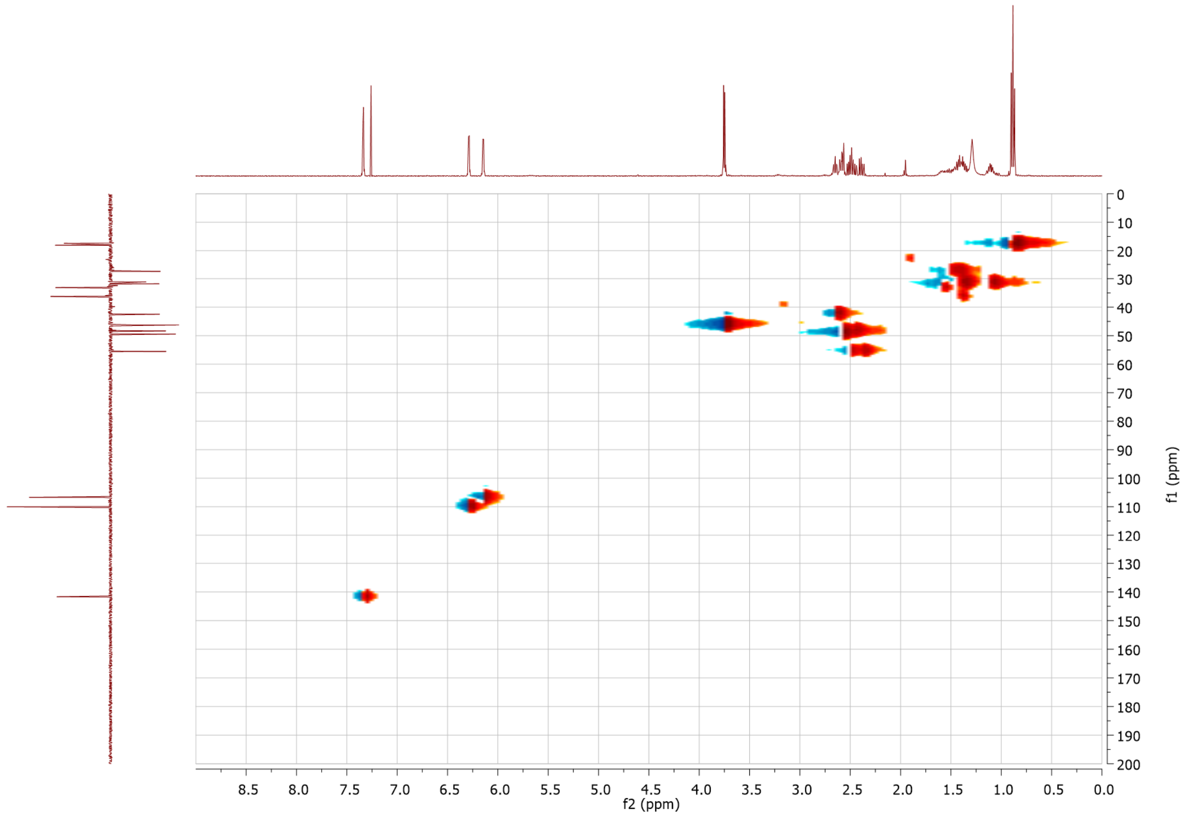

4. TGA measurements of each synthesized amines (under nitrogen, at 20 K.min^{-1})

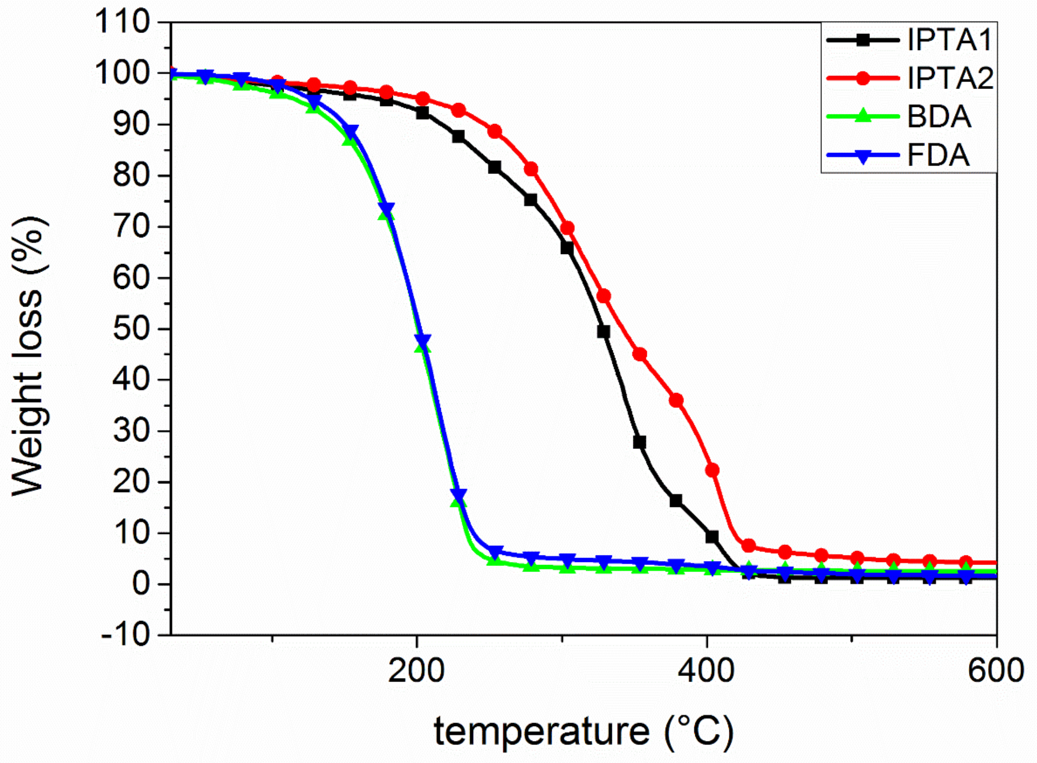

5. DSC measurements of each synthesized amines (under nitrogen, at 20 K.min-1)

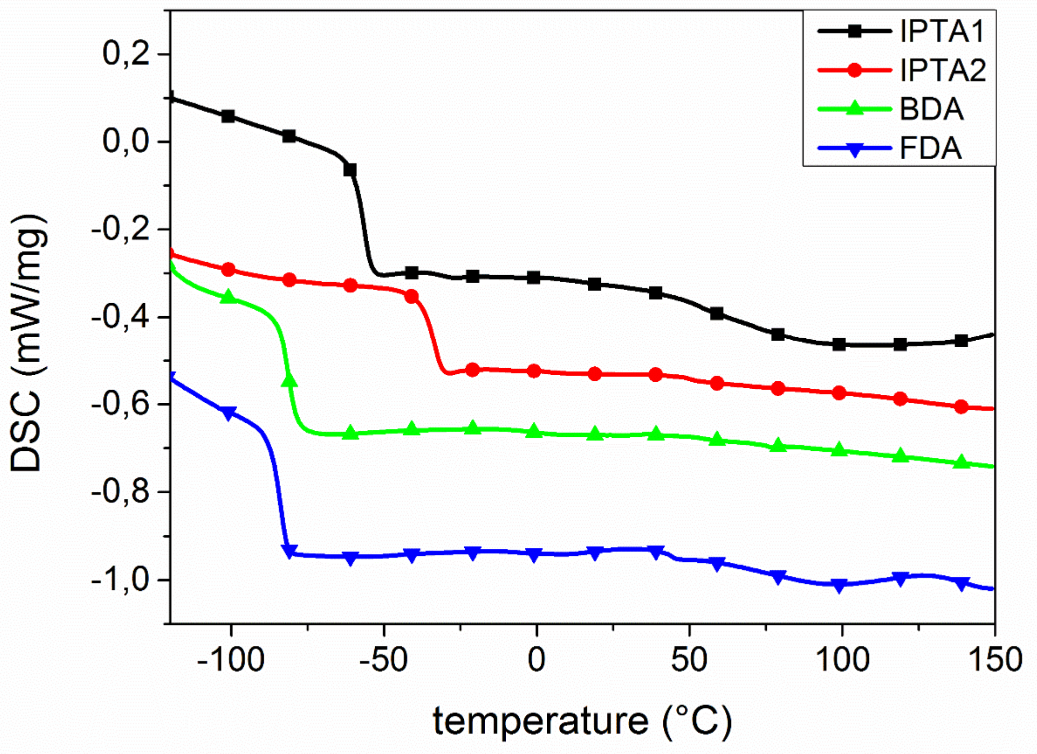

6. Determination of optimal ratio for DGEBA-based thermosets

a. From IPTA1 and DGEBA

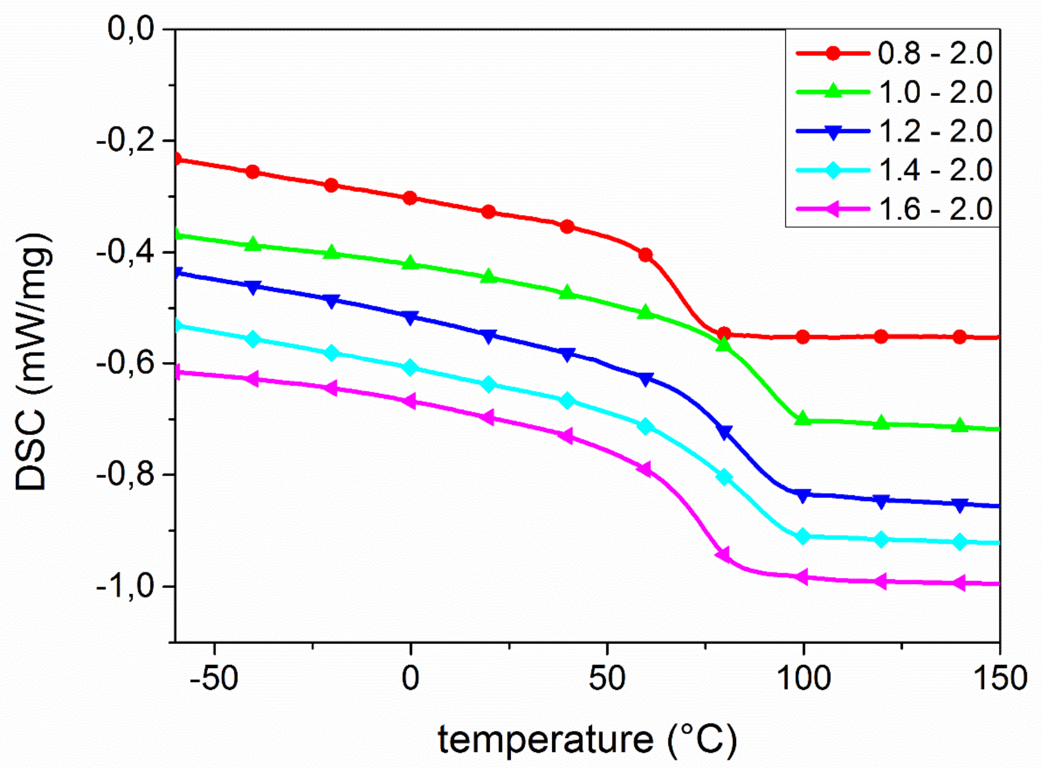

b. From IPT2 and DGEBA

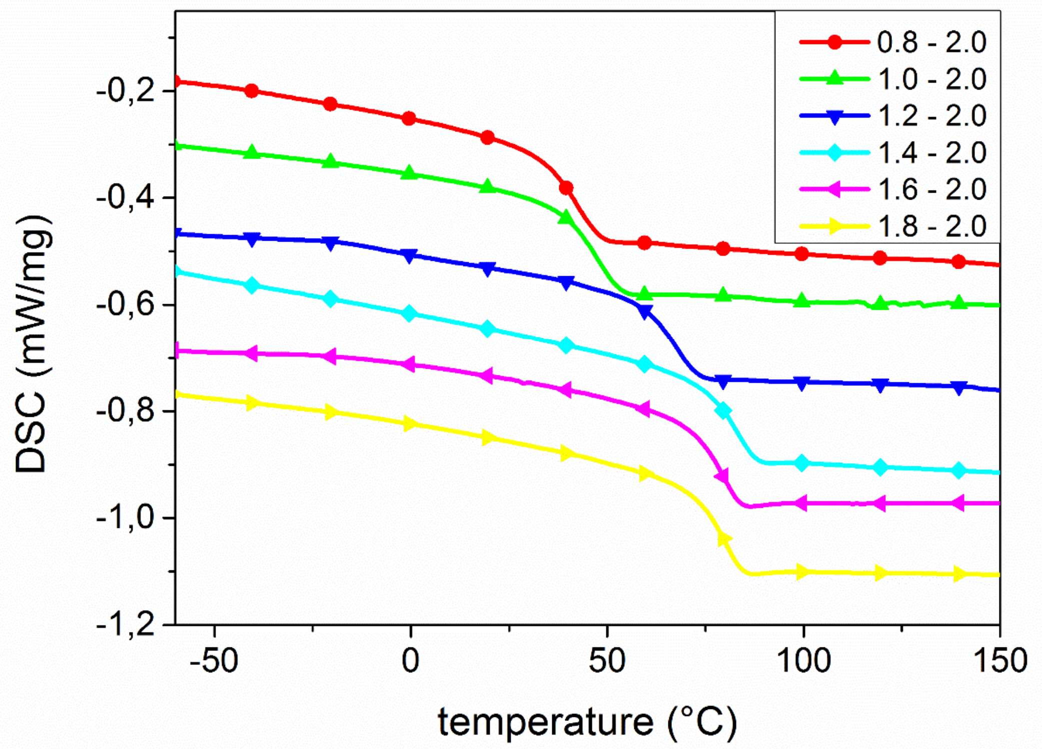

c. From BDA and DGEBA

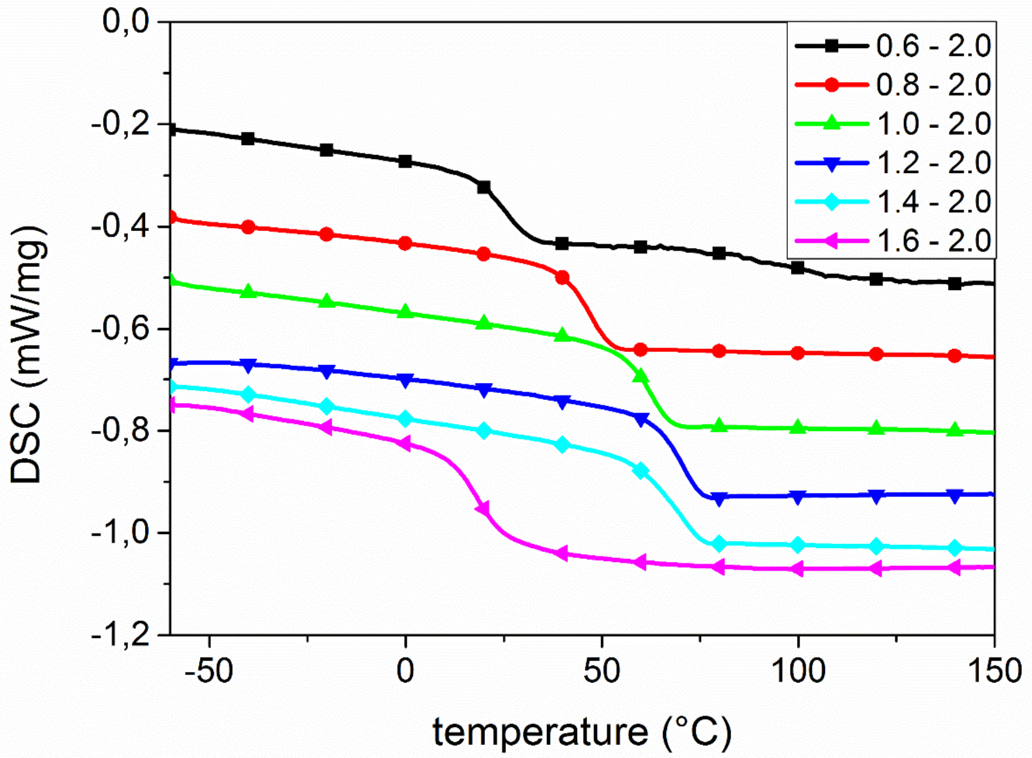

d. From FDA and DGEBA

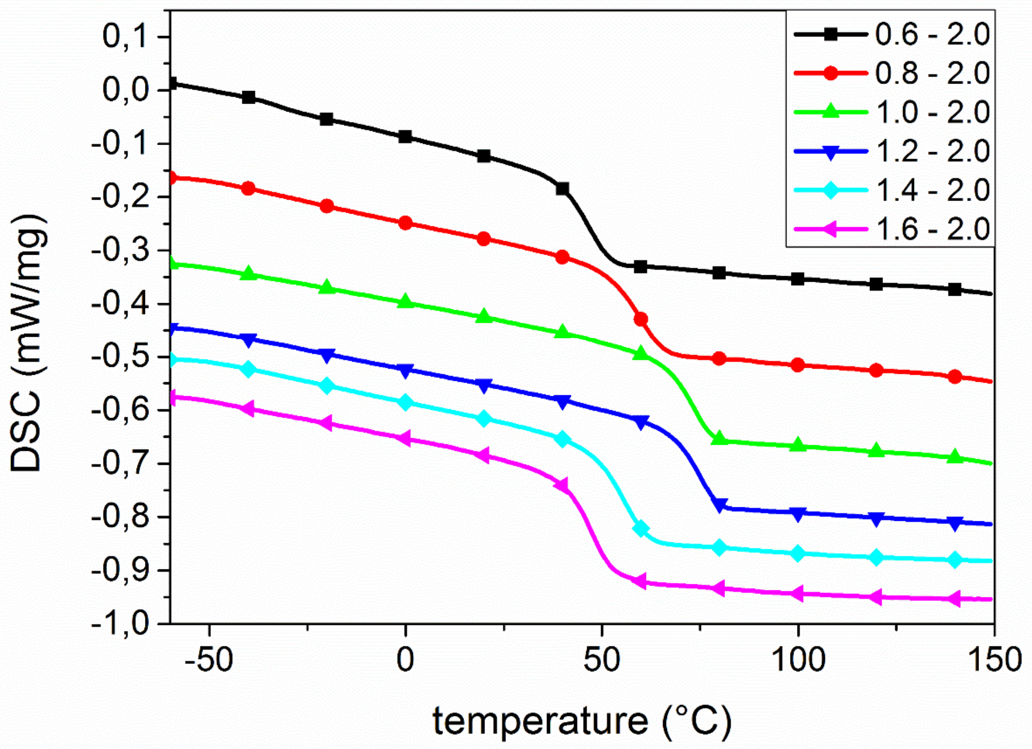

7. Determination of optimal ratio DGEVA-based thermosets

a. From IPTA1 and DGEVA

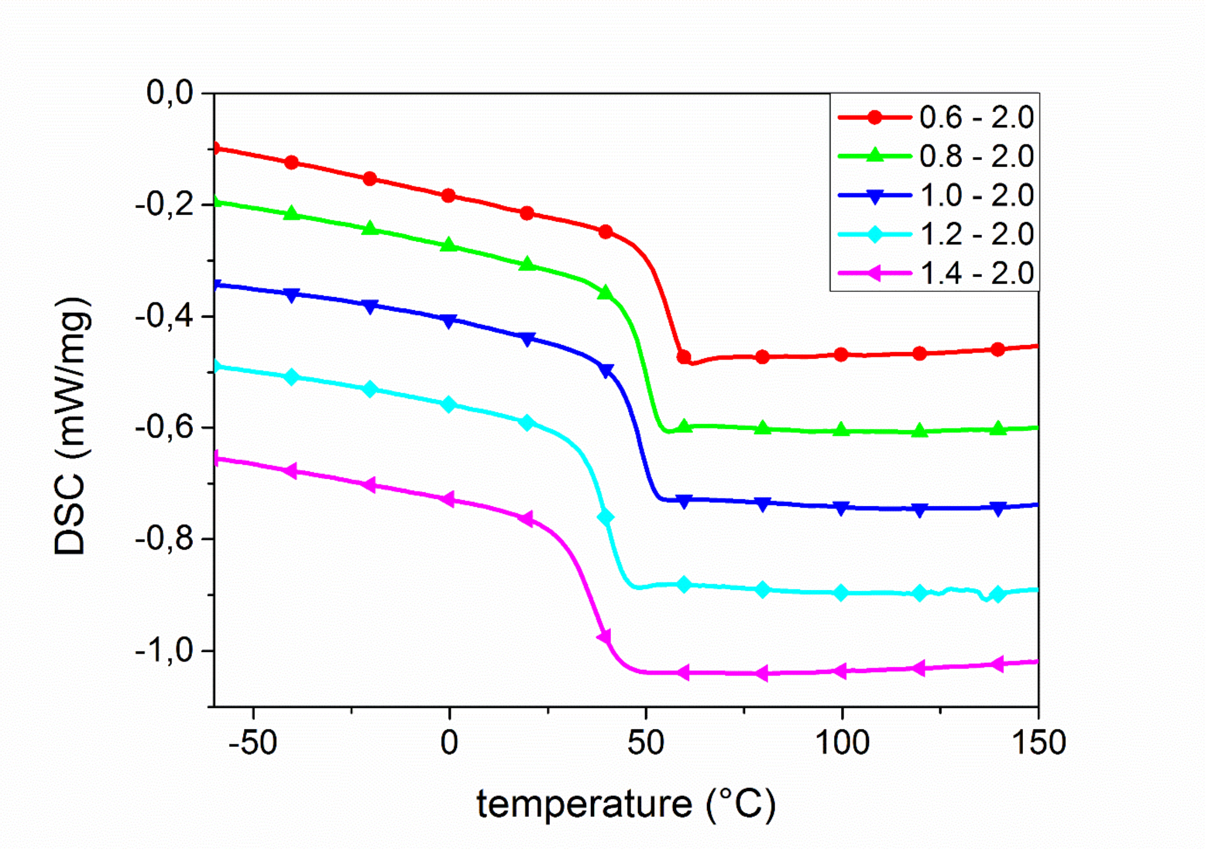

b. From IPTA2 and DGEVA

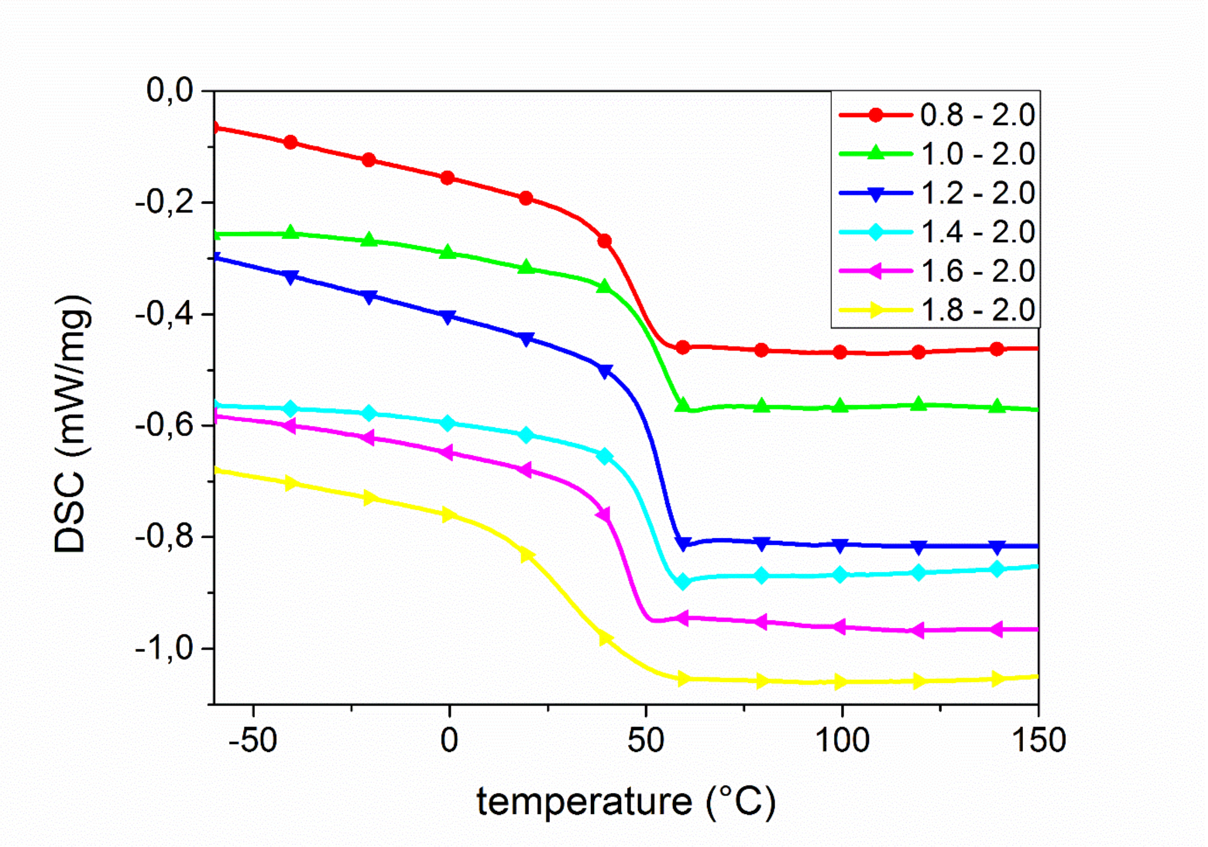

c. From BDA and DGEVA

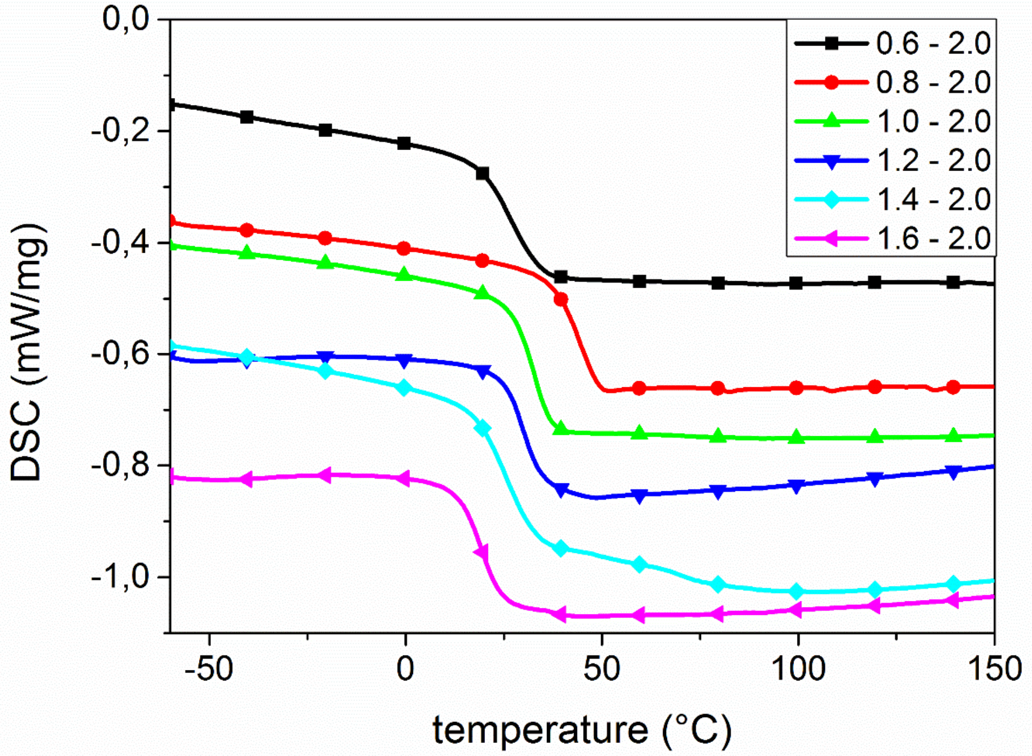

d. From FDA and DGEVA

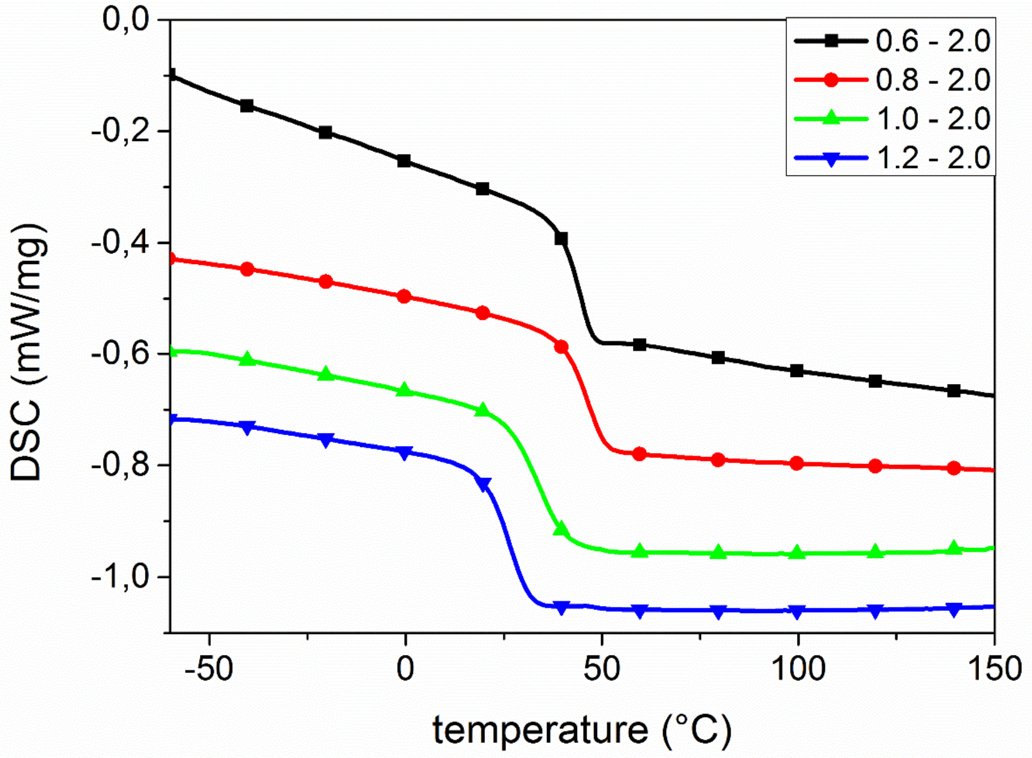

8. Characterizations of each bulk materials synthesized with optimal ratio

a. DSC of partially bio-based thermosets

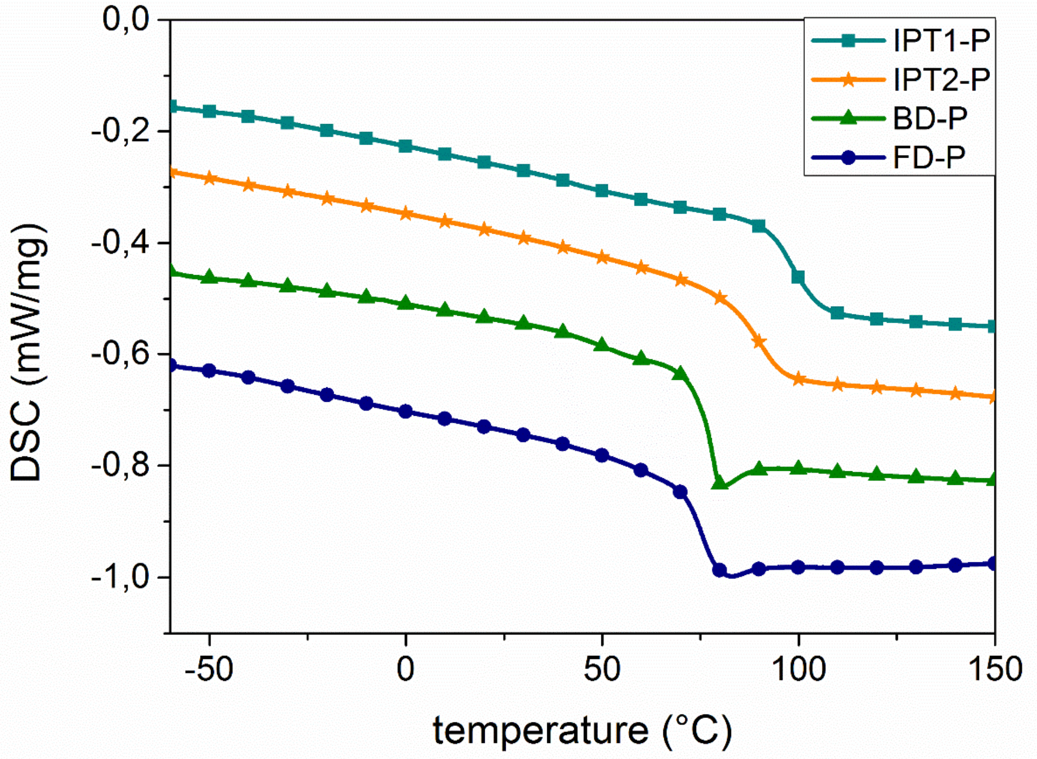

b. DSC of fully bio-based thermosets

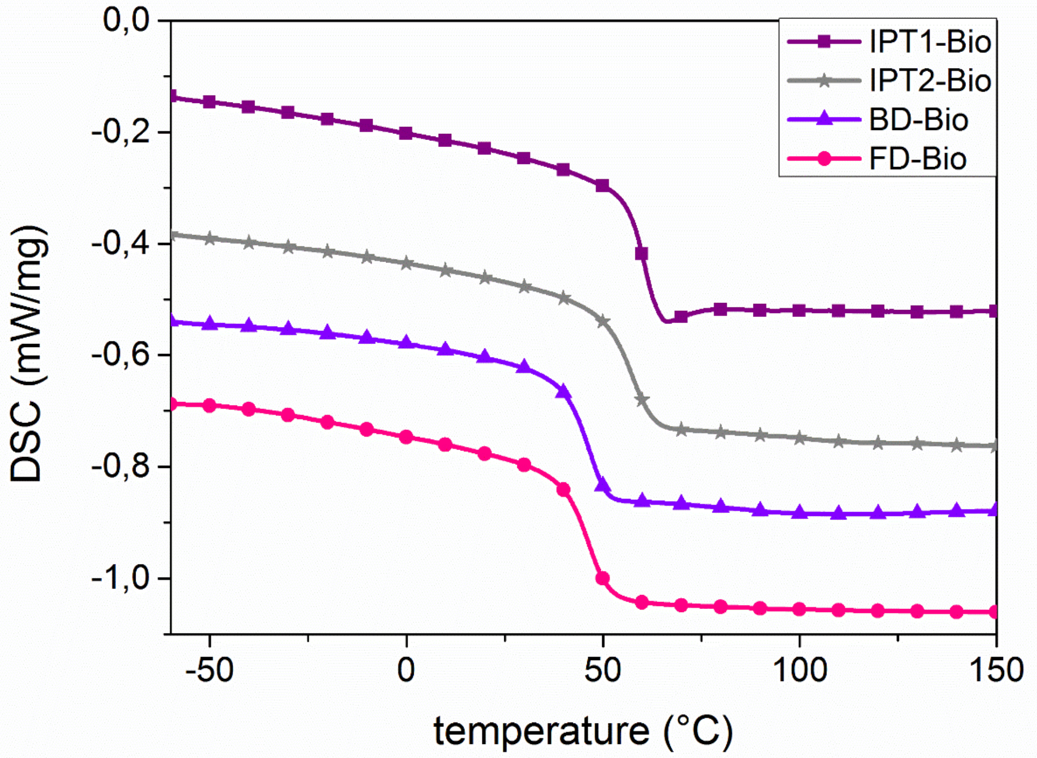

c. FT-IR of partially bio-based thermosets

i. IPT1-P

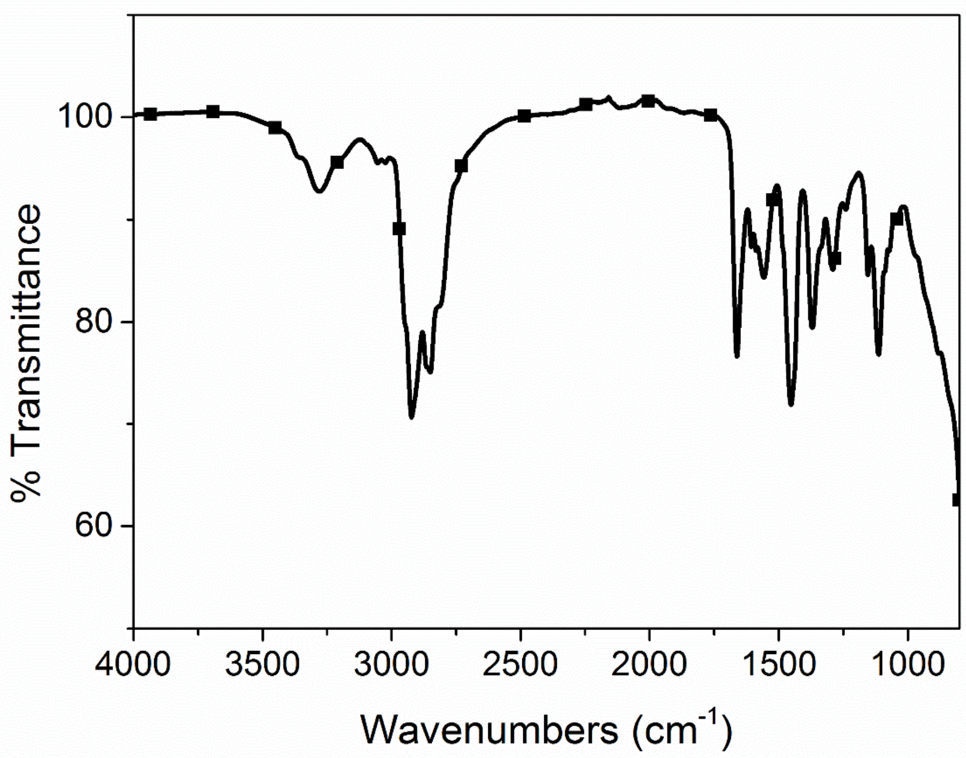

ii. IPT2-P

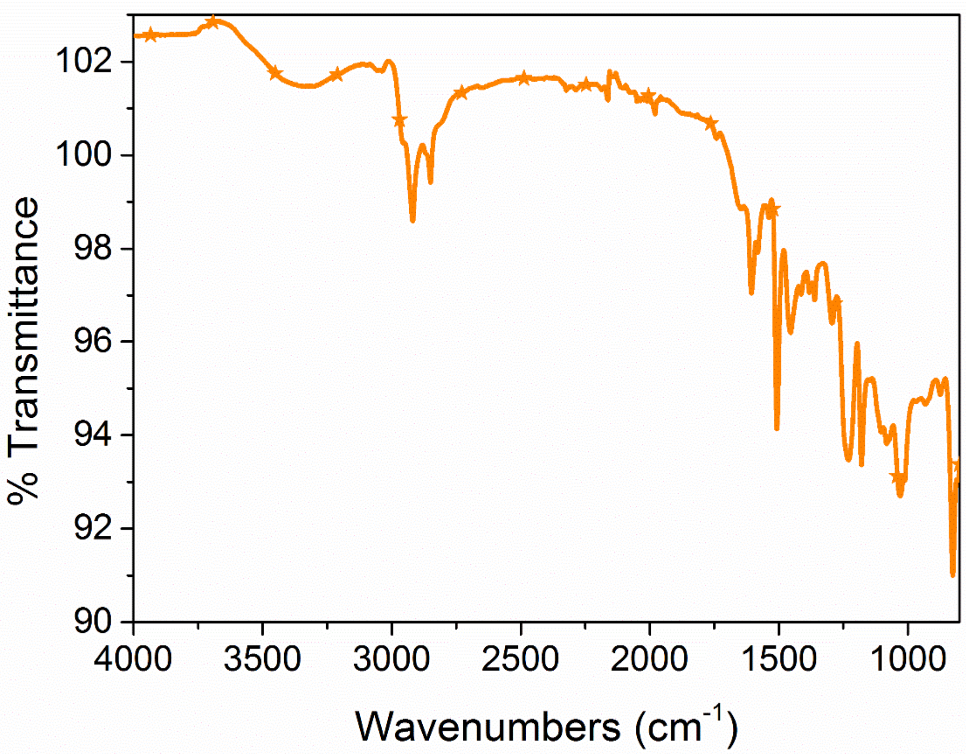

iii. BD-P

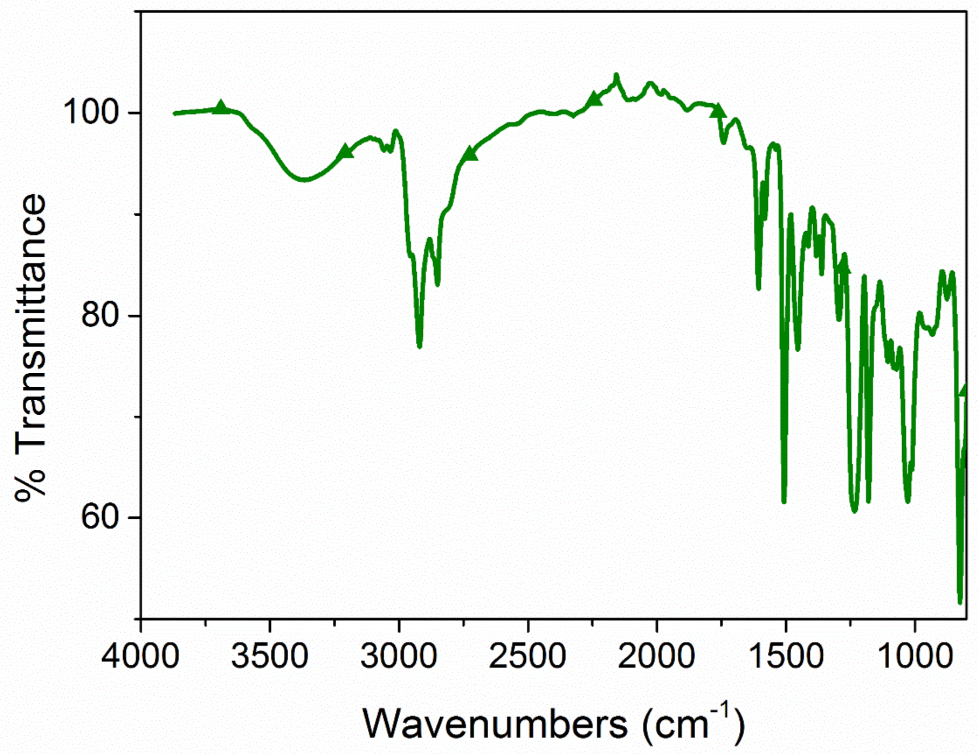

iv. FD-P

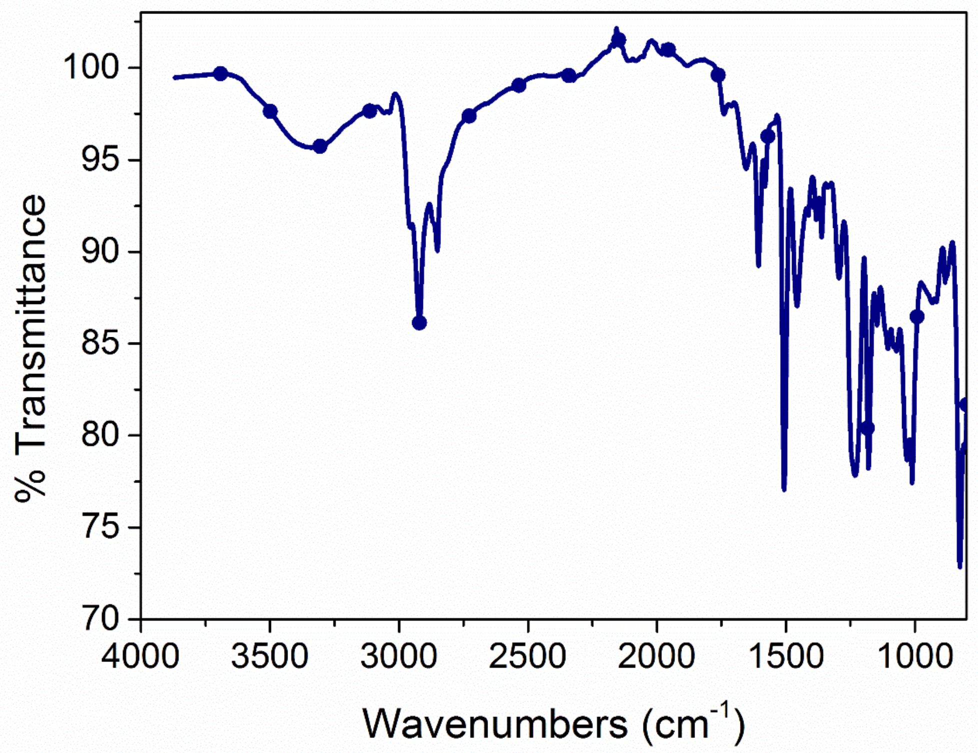

v. IPT1-Bio

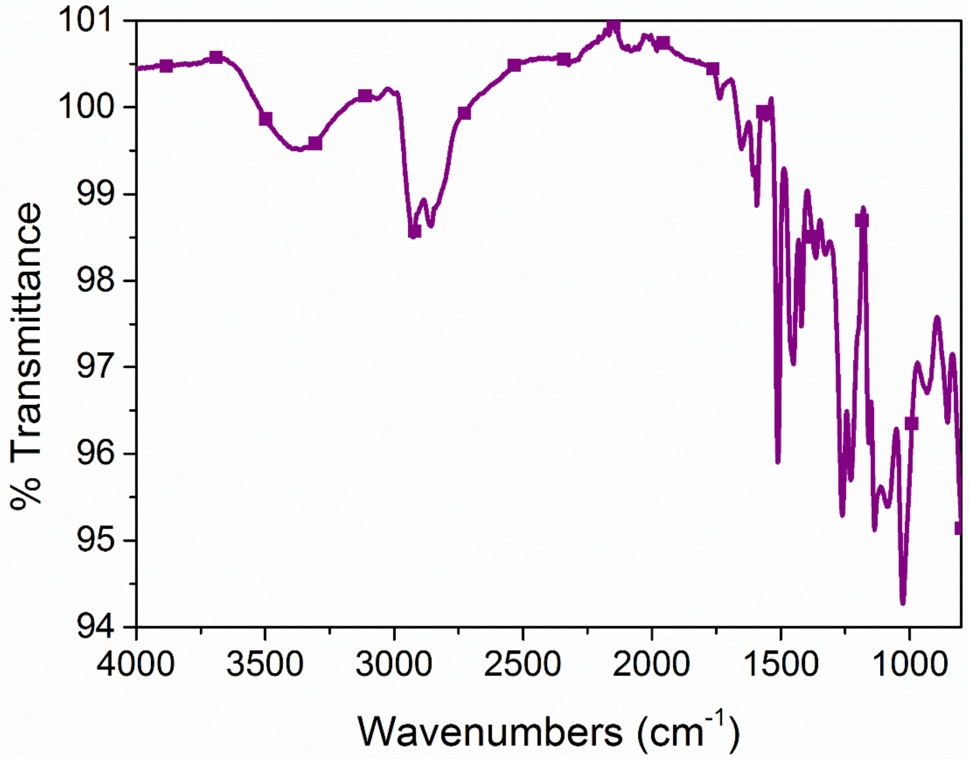

vi. IPT2-Bio

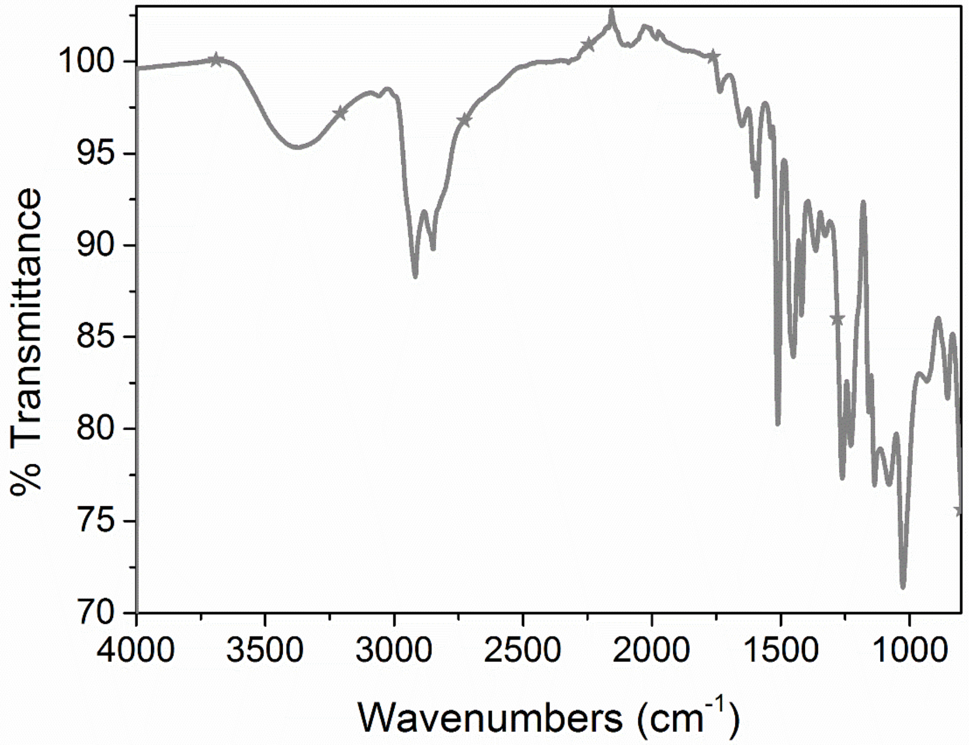

vii. BD-Bio

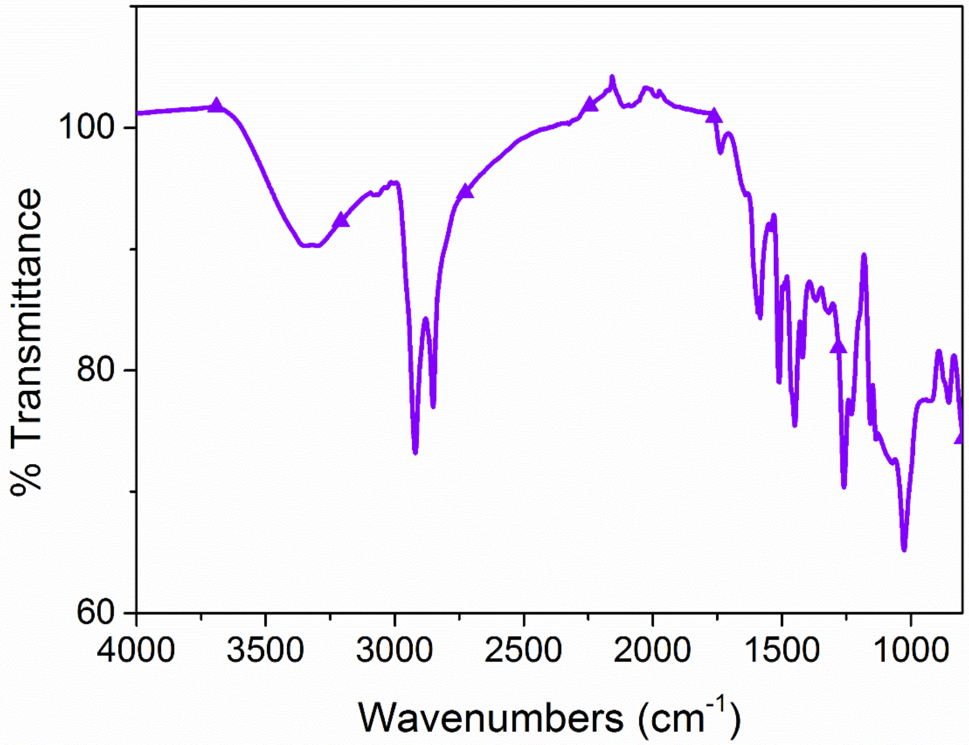

viii. FD-Bio

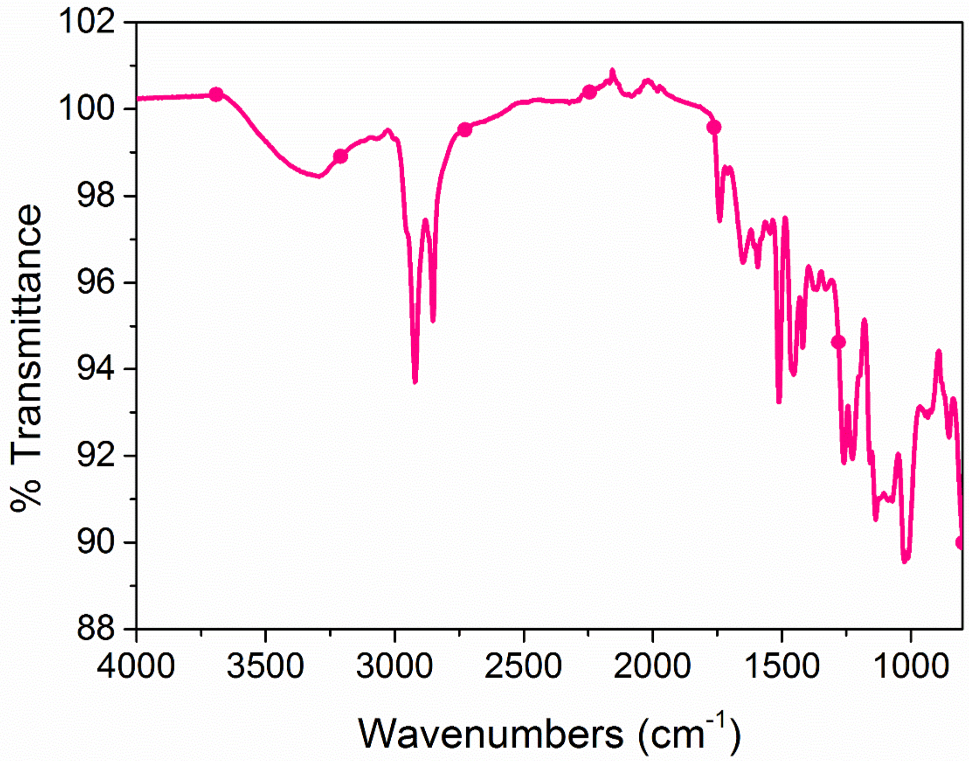

Supplement: Supplementary file 1 [file molecules-24-03285-s001.pdf]
